# Assessing the response of forest productivity to climate extremes in Switzerland using model–data fusion

**DOI:** 10.1111/gcb.15011

**Published:** 2020-02-18

**Authors:** Volodymyr Trotsiuk, Florian Hartig, Maxime Cailleret, Flurin Babst, David I. Forrester, Andri Baltensweiler, Nina Buchmann, Harald Bugmann, Arthur Gessler, Mana Gharun, Francesco Minunno, Andreas Rigling, Brigitte Rohner, Jonas Stillhard, Esther Thürig, Peter Waldner, Marco Ferretti, Werner Eugster, Marcus Schaub

**Affiliations:** ^1^ Department of Environmental Systems Science Institute of Agricultural Sciences ETH Zurich Zurich Switzerland; ^2^ Swiss Federal Institute for Forest, Snow and Landscape Research WSL Birmensdorf Switzerland; ^3^ SwissForestLab Birmensdorf Switzerland; ^4^ Faculty of Forestry and Wood Sciences Department of Forest Ecology Czech University of Life Sciences Prague Prague Czech Republic; ^5^ Theoretical Ecology University of Regensburg Regensburg Germany; ^6^ INRAE Aix‐Marseille Université UMR RECOVER Aix‐en‐Provence France; ^7^ W. Szafer Institute of Botany Polish Academy of Sciences Krakow Poland; ^8^ Department of Environmental Systems Science Institute of Terrestrial Ecosystems ETH Zurich Zurich Switzerland; ^9^ Department of Forest Sciences University of Helsinki Helsinki Finland

**Keywords:** Bayesian inference, carbon cycling, data assimilation, drought, ecosystem productivity, extreme events, *Fagus sylvatica*, inverse modeling, model calibration, *Picea abies*

## Abstract

The response of forest productivity to climate extremes strongly depends on ambient environmental and site conditions. To better understand these relationships at a regional scale, we used nearly 800 observation years from 271 permanent long‐term forest monitoring plots across Switzerland, obtained between 1980 and 2017. We assimilated these data into the 3‐PG forest ecosystem model using Bayesian inference, reducing the bias of model predictions from 14% to 5% for forest stem carbon stocks and from 45% to 9% for stem carbon stock changes. We then estimated the productivity of forests dominated by *Picea abies* and *Fagus sylvatica* for the period of 1960–2018, and tested for productivity shifts in response to climate along elevational gradient and in extreme years. Simulated net primary productivity (NPP) decreased with elevation (2.86 ± 0.006 Mg C ha^−1^ year^−1^ km^−1^ for *P. abies* and 0.93 ± 0.010 Mg C ha^−1^ year^−1^ km^−1^ for *F. sylvatica*). During warm–dry extremes, simulated NPP for both species increased at higher and decreased at lower elevations, with reductions in NPP of more than 25% for up to 21% of the potential species distribution range in Switzerland. Reduced plant water availability had a stronger effect on NPP than temperature during warm‐dry extremes. Importantly, cold–dry extremes had negative impacts on regional forest NPP comparable to warm–dry extremes. Overall, our calibrated model suggests that the response of forest productivity to climate extremes is more complex than simple shift toward higher elevation. Such robust estimates of NPP are key for increasing our understanding of forests ecosystems carbon dynamics under climate extremes.

## Graphical abstract

We assimilated extensive and long‐term monitoring data into the 3‐PG forest ecosystem model to assess how forest productivity responds to climate extremes across environmental gradients in Switzerland. Data assimilation using Bayesian inference significantly improved model predictions. During warm–dry extremes, net primary productivity increased at higher and decreased at lower elevations, with reductions in NPP of more than 25% for one‐fifth of the potential species distribution range in Switzerland.

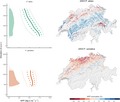

## 1 INTRODUCTION

Forests provide a wide range of ecosystem functions and services from global to local scale (Brockerhoff et al., 2017). It is therefore essential to understand how forest ecosystem productivity responds to climate extremes across environmental gradients (Ciais et al., 2014; Cramer et al., 2001; Reichstein et al., 2013) and how those responses feed back to the climate system (Humphrey et al., 2018). Climate change can affect forests on various levels, for example, by modifying the balance and interactions between direct abiotic constraints on tree growth (Cuny et al., 2019), shifting the timing of the growing season (Bigler & Bugmann, 2018), or altering disturbance regimes (Senf et al., 2018; Sommerfeld et al., 2018). Large‐scale variations in forest ecosystem productivity have been primarily attributed to interactions between environmental constraints, namely temperature, water availability and demand and radiation (Beer et al., 2010; Jung et al., 2017; Seddon, Macias‐Fauria, Long, Benz, & Willis, 2016), rather than to a single one. In particular, global warming amplifies water limitation as a key constraint for global forest ecosystem productivity, and the spatial extent of drought‐limited areas is increasing (Allen et al., 2010; Babst et al., 2019; D'Orangeville et al., 2018; Nemani et al., 2003).

A diverse set of methods is currently used to quantify and project the impact of changing environmental constraints on forest ecosystem productivity, including extensive collections of in situ observations (Babst et al., 2019; Charney et al., 2016; Clark et al., 2001; Klesse et al., 2018; Shestakova et al., 2019), remote sensing data (Beer et al., 2010; Jolly, Dobbertin, Zimmermann, & Reichstein, 2005; Nemani et al., 2003; Piao et al., 2014), or dynamic vegetation models (DVMs, e.g., Huang, Gerber, Huang, & Lichstein, 2016; Rollinson et al., 2017; Zhang et al., 2018). These and other studies identified important differences in the response of forests to environmental constraints, depending on ambient climate conditions. Forests growing in cold environments at high elevations and latitudes may benefit from higher temperatures because their productivity is predominantly limited by temperature and particularly by a short growing season. In contrast, forests at lower elevations may increasingly suffer from lack of soil water because warming causes an increase in atmospheric water demand, even if precipitation does not decrease (Körner & Paulsen, 2004). Accordingly, a recent research focus has been on warm and dry extremes, whereas cold and wet extreme events (e.g., Figure S1) have received less attention, despite their importance (but see Chen et al., 2019; Vitasse et al., 2019). Moreover, the quantification of forest ecosystem productivity responses to climate extremes at high spatial and temporal resolution is still rare.

A challenge in this context is the synthesis of various and often heterogeneous datasets into a product that summarizes our best knowledge about the dynamics and climate sensitivities of forest ecosystems and that can be used for projections. DVMs can achieve this purpose, especially when coupled with various types of monitoring data (Hartig et al., 2012). We refer to this process as data assimilation (also “model–data fusion” or "inverse modeling"; see Keenan, Davidson, Moffat, Munger, & Richardson, 2012). Data assimilation can help to better estimate the true ecosystem state, its dynamics, and the associated uncertainties (Keenan, Carbone, Reichstein, & Richardson, 2011; Lahoz, Khattatov, & Menard, 2010; Niu et al., 2014). Data assimilation can also reduce uncertainties in many areas of the modeling process, for example, via initial state updating (data assimilation in a narrow sense), parameter estimation (model–data fusion, model calibration), input updating, and error correction (Houser, De Lannoy, & Walker, 2010). Recent research in forest ecosystem sciences emphasizes parameter estimation techniques to better constrain DVMs (LeBauer, Wang, Richter, Davidson, & Dietze, 2013; Luo et al., 2011; MacBean, Peylin, Chevallier, Scholze, & Schürmann, 2016; Peng, Guiot, Wu, Jiang, & Luo, 2011; Scholze, Buchwitz, Dorigo, Guanter, & Quegan, 2017), which has led to the development of online and offline data assimilation systems (Anderson et al., 2009; Dietze, Lebauer, & Kooper, 2013; Huang et al., 2019; Peylin et al., 2016). Parameter estimation via data assimilation helps to estimate the statistical distribution of model parameter values such that model outputs better reflect the currently available information (Hartig et al., 2012; Huang et al., 2019). Bayesian inference has often been recommended as the most useful technique to achieve these goals (Hartig, Dislich, Wiegand, & Huth, 2014; van Oijen, 2017). Despite the fact that this method allows us to combine multiple data sources and types, most studies have focused on the local scale. Hence, an important step forward is now to use large and diverse datasets in combination with DVMs at the regional scale (Cailleret, Bircher, Hartig, Hülsmann, & Bugmann, 2019; Fer et al., 2018; Minunno, Peltoniemi, et al., 2019; Thomas et al., 2017; Van Oijen et al., 2013).

We assimilated extensive and long‐term forest ecosystem monitoring data into the 3‐PG forest ecosystem model (Landsberg & Waring, 1997). With the parameterized model, we assessed how forest productivity responds to climate extremes across environmental gradients in Switzerland. Switzerland is a highly suitable case study for this purpose, because its elevational gradients span a range of bioclimatic conditions that are comparable to at least 1,800 km of latitudinal gradient in Europe (Halbritter, Alexander, Edwards, & Billeter, 2013), but within a small geographic area. This alleviates the need to control for different synoptic drivers, continentality, population genetic differences, etc. To constrain the parameter distributions of 3‐PG and estimate their uncertainty ranges, we compiled monitoring data for two dominant European species (*Picea abies* (L.) H. Karst. and *Fagus sylvatica* L.) from 271 sites, totaling almost 800 observation years. We then used the constrained model parameter distributions to test for shifts in forest productivity responses to climate extremes across environmental gradients. Specifically, we addressed the following questions: (a) What is the contrast in climate response at low versus high elevation and in average versus extreme years? (b) How strong are NPP anomalies during warm versus cold extremes and what is the spatial extent of the affected area? Answering these questions helps us to better understand and anticipate possible trajectories of forest ecosystem productivity in a warmer and more variable future climate.

## 2 MATERIALS AND METHODS

### 2.1 Monitoring data

**Figure 1:**
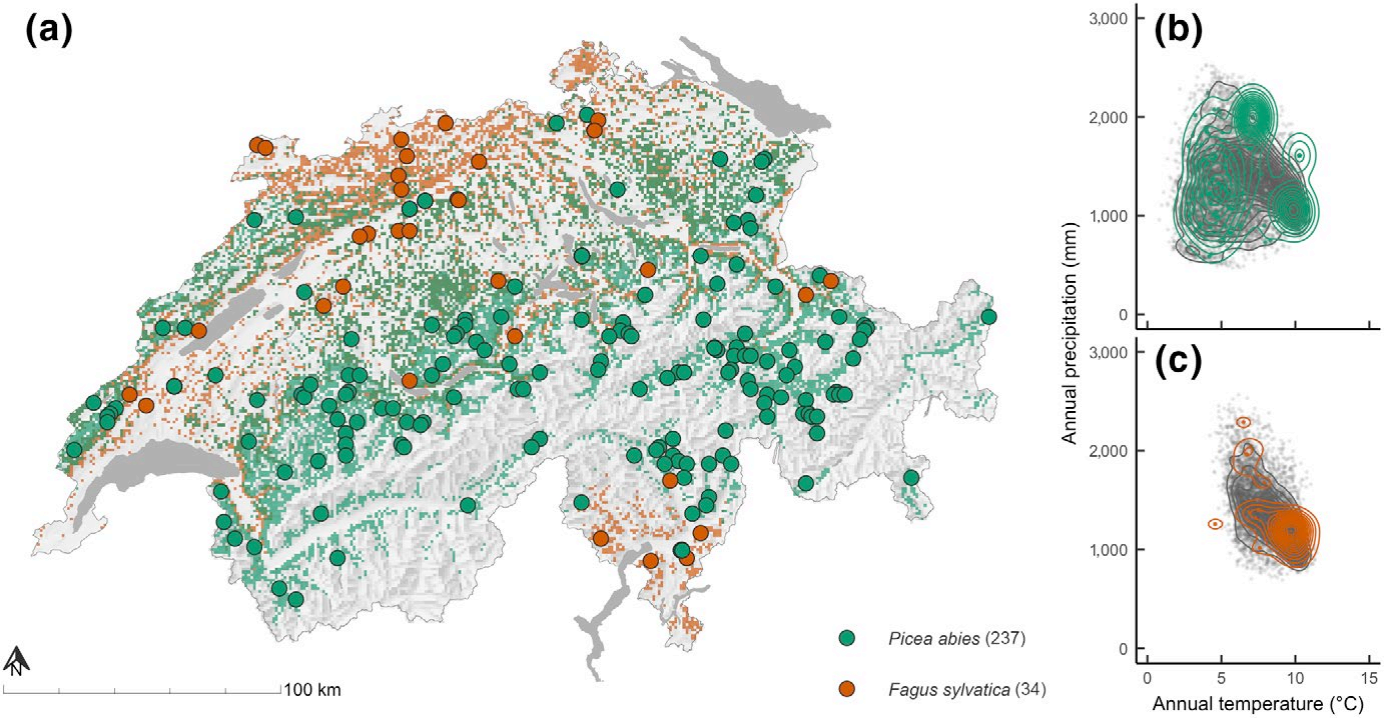
Location of the 271 monitoring plots (dots) distributed across the potential habitats (colored background) of *Picea abies* and *Fagus sylvatica* dominated forests in Switzerland (a). Potential habitats are based on the MoGLI projections (Wüest et al., 2020). (b, c) Distribution of the selected plots (colored dots and contours) compared to all potential habitats in Switzerland (gray dots and contours) along the annual mean temperature and annual precipitation sum gradients

We used data from 271 permanent forest monitoring plots covering the actual habitat of *P. abies* (*N* = 237) and *F. sylvatica* (*N* = 34; Figure 1) across Switzerland. The datasets cover the period from 1980 to 2017 and include selected plots from the Swiss National Forest Inventory (NFI; Fischer & Traub, 2019), the Experimental Forest Management (EFM) network (Forrester, Nitzsche, & Schmid, 2019), the Long‐term Forest Ecosystem Research Network (LWF; Etzold, Waldner, Thimonier, Schmitt, & Dobbertin, 2014; Schaub, Dobbertin, Kräuchi, & Dobbertin, 2011; Thimonier et al., 2010), and one forest site from the Swiss FluxNet (Etzold et al., 2011; Zielis et al., 2014). We used eight variables that describe stand stocks and characteristics: stem biomass (SB), foliage biomass, root biomass, number of trees, average diameter at breast height (1.3 m; DBH), basal area (BA), leaf area index (LAI), and gross primary production (GPP). To calculate the stand‐level stocks, we applied the biomass equations developed for European forests following Forrester, Tachauer, et al. (2017) for each measured tree, and summed it up to the stand level in Mg dry matter/ha. The first observations on each monitoring plot were used to initialize the 3‐PG model runs (see below).

### 2.2 National Forest Inventory

The Swiss NFI records the current state of forests on a regular grid of 1.4 km covering about 6,500 permanent monitoring plots that have been measured since 1983 (Brändli, 2010; Fischer & Traub, 2019). Each plot is remeasured every 10 years, with a one‐time change in timing due to a switch from a periodic to a continuous survey in the fourth NFI phase (i.e., since 2009). The NFI plot design comprises nested circular plots, such that every tree with a DBH ≥ 12 cm is recorded within an inner 200 m^2^ circle (radius = 7.98 m), and every tree with a DBH ≥ 36 cm is recorded within a 500 m^2^ circle (radius = 12.62 m). For every individual tree, the position, DBH, status, and species are recorded. In addition, tree height (*H*) and crown length (*H*_c_) are measured on a subset of trees. Age is estimated based on a regression model that was fit to the data obtained either from counting tree rings or counting layers of whorling branches (for the *P. abies* trees) directly on the plot (Brassel & Lischke, 2001). Management and mortality on each individual monitoring plot were derived from inventory data. The specific year of management interventions and the timing of tree mortality were randomly assigned between two consecutive inventories, as the exact dates are unknown. The monitoring plots for this study were selected using the following criteria: (a) monospecific even‐aged stands of either *P. abies* or *F. sylvatica*, (b) at least two consecutive remeasurements were available, (c) no ingrowth during the selected period, (d) no obvious measurement errors or missing measurement, and (e) stand age estimation was available for the first observation used. Based on these criteria, we retained 176 (*P. abies* *N* = 147, *F. sylvatica* *N* = 29) NFI plots, in total accounting for 451 observation years. The time span between the first and the last measurement ranged between 4 and 35 years.

### 2.3 Experimental Forest Management

The EFM project has been collecting growth and yield data for more than a century (Forrester et al., 2019). The EFM network currently includes 459 permanent monitoring plots, which are measured every 5–12 years, depending on their growth rates, stand age, and research objectives. The EFM monitoring plots are of varying size with precisely defined boundaries, within which all individual trees with a DBH ≥ 8 cm are measured. For each tree, the position, DBH, status, and species are recorded. In addition, *H* and *H*_c_ are measured for a subset of trees. Age is estimated based on the planting date in even‐aged stands. Management (thinning type and timing) is recorded for each individual monitoring plot and is done in the same year as the measurements. The monitoring plots for this study were selected based on the same criteria used for NFI monitoring plots. Based on those criteria, 94 plots remained in our analysis (*P. abies* *N* = 89, *F. sylvatica* *N* = 5), in total accounting for 331 observation years. The time span between the first and the last measurement ranged between 5 and 30 years.

### 2.4 Long‐term Forest Ecosystem Research and Swiss FluxNet

In the LWF network, information on tree growth and crown condition as well as on the nutrient cycle and the ecosystem water balance is collected to assess the impact of environmental changes on forest functioning (Etzold et al., 2014; Schaub et al., 2011; Thimonier et al., 2010). The LWF network includes 19 permanent monitoring plots, on which monitoring data have been recorded every 1–5 years, since 1994. The LWF uses a standardized protocol for data collection based on the International Co‐operative Programme on Assessment and Monitoring of Air Pollution Effects on Forests (UNECE ICP FOrests Programme Co‐ordinating Centre, 2016). The monitoring plots for this study were selected based on the same criteria used for the NFI and EFM plots and one plot remained (*P. abies* *N* = 1). The retained LWF *P. abies* plot (Davos CH‐Dav) is also part of the Swiss FluxNet ecosystem‐scale CO_2_ and H_2_O vapor eddy‐covariance flux measurement network (Etzold et al., 2011), providing measurements of net ecosystem production, GPP, and ecosystem respiration.

### 2.5 Dynamic vegetation model

3‐PG is a process‐based forest ecosystem model that consists of five submodels in a causal chain, starting with light absorption and assimilation, and ending with the conversion of biomass into output variables (Landsberg & Waring, 1997; Sands & Landsberg, 2002). A simple structure, readily obtainable input data, and a low number of parameters have facilitated the widespread use of 3‐PG in various forest types around the world (Gupta & Sharma, 2019). Initially developed for simulating evergreen, even‐aged, monospecific forests, the model has recently been further developed for deciduous, uneven‐aged, and mixed‐species forests (Forrester & Tang, 2016). It is a cohort‐based, non‐spatially explicit model with a monthly time step. Each cohort can be a different species and/or age class. Stand‐level calculations avoid the propagation of potential errors when scaling up from higher resolution calculations (e.g., leaves or trees) while providing outputs at the level required for this study (Landsberg & Waring, 1997; Pretzsch, Forrester, & Rötzer, 2015).

The first of the five submodels predicts light absorption and GPP using a species‐specific canopy quantum efficiency (*α*_C_). The *α*_C_ is reduced in response to limitations imposed by temperature, frost, vapor pressure deficit (VPD), soil moisture, soil nutrient status, atmospheric CO_2_, and stand age (Almeida, Landsberg, & Sands, 2004; Landsberg & Waring, 1997; Sands & Landsberg, 2002). NPP is calculated as a fixed fraction of GPP (Waring, Landsberg, & Williams, 1998) and is distributed to roots, stems, and foliage by the second submodel. Partitioning to aboveground versus belowground biomass depends on soil nutrient status, VPD, and soil moisture, while partitioning between stems and foliage depends on tree size, with larger trees partitioning a lower proportion of NPP to foliage compared to smaller trees (Landsberg & Waring, 1997; Sands & Landsberg, 2002). The third submodel simulates density‐dependent mortality, which is calculated using the −3/2 self‐thinning law by Yoda (1963). In the fourth submodel, which calculates the water balance, the Penman–Monteith equation is used to calculate transpiration and soil evaporation, which are added to canopy interception to predict evapotranspiration. Canopy conductance *g*_c_ is determined using a species‐specific maximum *g*_c_, LAI and limitations caused by VPD, soil moisture, atmospheric CO_2_, and stand age. Changes in soil water storage are calculated as the difference between evapotranspiration and rainfall; any excess of the maximum soil water holding capacity is drained off (Sands & Landsberg, 2002). The fifth submodel converts biomass into output variables such as mean tree diameter, height, BA, wood volume, etc., using allometric relationships. Different management strategies are specified using the residual stocking (trees/ha) after thinning at a nominal age. Thinning from below or above is achieved by specifying the fraction of the foliage, root, and SB of an average tree that was thinned (Landsberg, Mäkelä, Sievänen, & Kukkola, 2005). All submodels were evaluated by comparing predictions of the given process against empirical data of that process for many different forest types (Gupta & Sharma, 2019; Landsberg & Sands, 2011), including central European forests (Forrester, Ammer, et al., 2017; Nolè et al., 2009).

For our simulations, we used a re‐implementation of the 3‐PG model programmed in Fortran 90 (Minunno, Hartig, & Trotsiuk, 2019). It was driven with time series monthly mean of daily minimum and maximum temperatures (*T*_min_, *T*_max_, °C), rainfall (*P*_rcp_, mm/month), monthly mean of daily solar radiation (*S*_rad_, MJ m^−2^ day^−1^), and the number of frost days (*F*_days_, days/month with *T*_min_ < 0°C). We used spatially interpolated monthly meteorological data as input for 3‐PG (Figure S2). The interpolation (100 m spatial resolution) of the meteorological data was done by the Landscape Dynamics group (WSL, Switzerland) using data from MeteoSwiss stations (Swiss Federal Office of Meteorology and Climatology) by employing the DAYMET method (Thornton, Running, & White, 1997). Site‐specific information on soil type and plant available soil water was retrieved from a digitized soil suitability map of Switzerland (scale 1:200,000; Frei et al., 1980; Swiss Federal Statistical Office, 2000).

### 2.6 Parameter estimation

We used Bayesian inference to derive the parameter estimates and uncertainties of the 3‐PG model. The approach accounts for observational uncertainties and to make use of multiple types of data at different temporal scales. We assumed uniform (i.e., non‐informative) prior distributions for each of the 54 model parameters. The ranges of the priors (Tables S3 and S4) were set to the minimum (maximum) value found in the literature minus (or plus) half of the range for this parameter (following Augustynczik et al., 2017). The likelihood function was constructed to be robust against outliers by modeling the residual error as a Student's *t* distribution with sampled degrees of freedom (see Code S1; Lange, Little, & Taylor, 1989). We used the Differential Evolution Markov Chain Monte‐Carlo algorithm (DEzs MCMC, ter Braak & Vrugt, 2008), implemented in the *BayesianTools R* package (Hartig, Minunno, & Paul, 2019) to estimate the joint posterior distribution for the model parameters. For each species, we ran three independent DEzs MCMC runs, each with three internal chains, and tested convergence by visual inspection of the trace plots and additionally using the Gelman–Rubin diagnostic (Gelman & Rubin, 1992), with convergence being accepted when the multivariate potential scale reduction factor was ≤1.1. Three independent DEzs MCMC chains with 2.4 × 10^7^ (*P. abies*) and 1.7 × 10^7^ (*F. sylvatica*) iterations were required to achieve convergence. All analyses and calculations were performed in the *R* language for statistical computing (R Core Team, 2018).

### 2.7 Model evaluation and validation

To evaluate the skill of the model and generate model projections, we calculated posterior predictive distributions by running the model with 1,000 random samples from the parameters' posterior distribution. Model performance was evaluated using the percentage bias (pBias), root mean squared error (RMSE), and normalized root mean squared error (NRMSE). We first calculated statistics on the plot level, and then averaged over plots for each of the 1,000 samples. For the validation, we only used the most recent set of observations at all permanent monitoring plots to maximize the time between initialization and validation, which ranged from 4 to 35 years. To perform cross‐validation, we randomly split the full set of monitoring data into two equally sized groups, resulting in a calibration and a validation set.

### 2.8 Model simulations

We simulated forest productivity (i.e., NPP) for the species' potential distribution range in Switzerland (Wüest, Bergamini, Bollmann, & Baltensweiler, 2020) on a 1 × 1 km grid for a total of 10,100 grid points for *P. abies* and 7,030 grid points for *F. sylvatica*. For this purpose, we first simulated the growth of *P. abies* and *F. sylvatica* monocultures with the average climate observed during the 1961–1990 period, until the age of 40 years (spin‐up). The stands were simulated starting as 2‐year‐old plantations with an initial density of 10,000 trees/ha. Thinning was performed at age 20 and 35 to reach a final density of ca. 1,000 trees/ha at age 40. We then simulated 30 years forced by monthly resolved climatic data from either the 1961–1990 (reference, according to MeteoSwiss) or the 1991–2018 period. We neglected the first 40 years of simulations due to high variation in productivity caused by early stage stand development. To study the impact of climate extremes on NPP, we focused on the deviation in NPP (expressed in percentage difference from the reference period) during the 30 year period (age 41–70). Furthermore, we compared our results to those derived from a remote sensing approach using the NPP product from MODIS (MOD17A3.055; Running, Mu, & Zhao, 2011) for the 2000–2014 period. While we limited the MODIS grid to that of the species' potential distribution range in Switzerland (Wüest et al., 2020), we cannot ensure that only *P. abies* or *F. sylvatica* dominated forest stands were included. Based on NFI, only 57% (*P. abies*) and 32% (*F. sylvatica*) of the selected MODIS grid cells have a dominant species *P. abies* or *F. sylvatica*, respectively.

## 3 RESULTS

### 3.1 Parameter estimation

The width of the posterior (measured by the 95% quantile range) was on average 59% (*P. abies*) and 32% (*F. sylvatica*) smaller than the prior range across all parameters (Tables S3 and S4). The largest reduction in uncertainty for both species was for parameters associated with allometric relationships, biomass partitioning, and stem mortality. Monitoring data were least informative for the parameters associated with branch and bark fractions and soil fertility. The parameter *u*, controlling the number of degrees of freedom in the Student's *t* likelihood (code S1), was much smaller for the *F. sylvatica* compared to the *P. abies* monitoring plots, indicating heavier tails in the error for *P. abies* (Tables S3 and S4).

**Figure 2:**
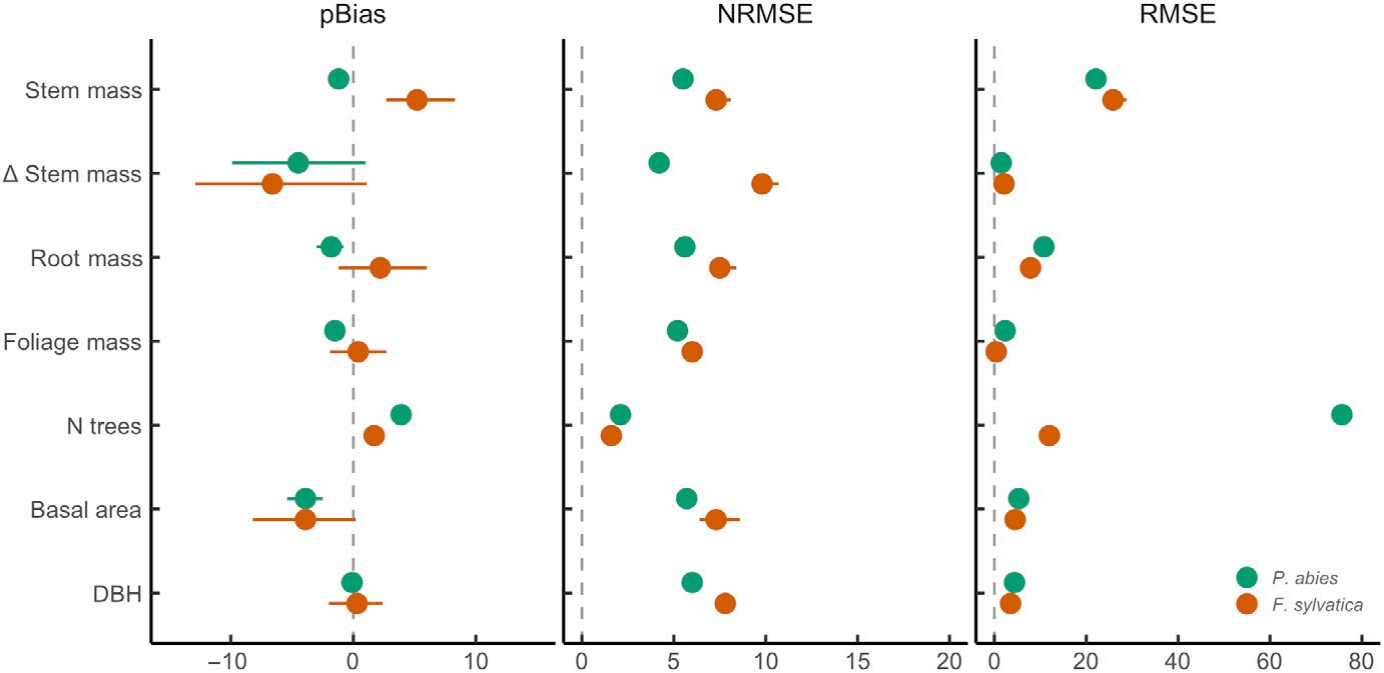
Statistics on predictive error (percent bias [pBias], normalized root mean squared error [NRMSE], and root mean squared error [RMSE]) of the 3‐PG model. The posterior predictive uncertainty was calculated by drawing 1,000 parameter combinations from the posterior distribution and calculating model predictions for these combinations. The dots represent the median value of the posterior predictive distribution, while the horizontal lines represent the 95% confidence interval. DBH, diameter at breast height

Predictions based on the posterior distributions significantly improved compared to predictions based on the prior distributions (Figure S3) for both *P. abies* and *F. sylvatica* (Figure 2). The NRMSE were below 8%, and the magnitude of the pBias was below 10% for all variables, while it reached up to 600% with the prior distributions (Figure S3). The correlations between observed and simulated values were high for all variables with *r* ^2^ ≥ .90 for *P. abies* and *r* ^2^ ≥ .87 for *F. sylvatica*. The RMSE for the change in stem dry biomass (ΔWS) of *P. abies* and *F. sylvatica* was 15 and 18 Mg/ha, respectively, while pBias was −7% and −9%, respectively (Figure 2). The cross‐validation based on 50% of all plots confirmed the high accuracy of the model (Figure S4).

### 3.2 Simulations of net primary productivity at the country scale

**Figure 3:**
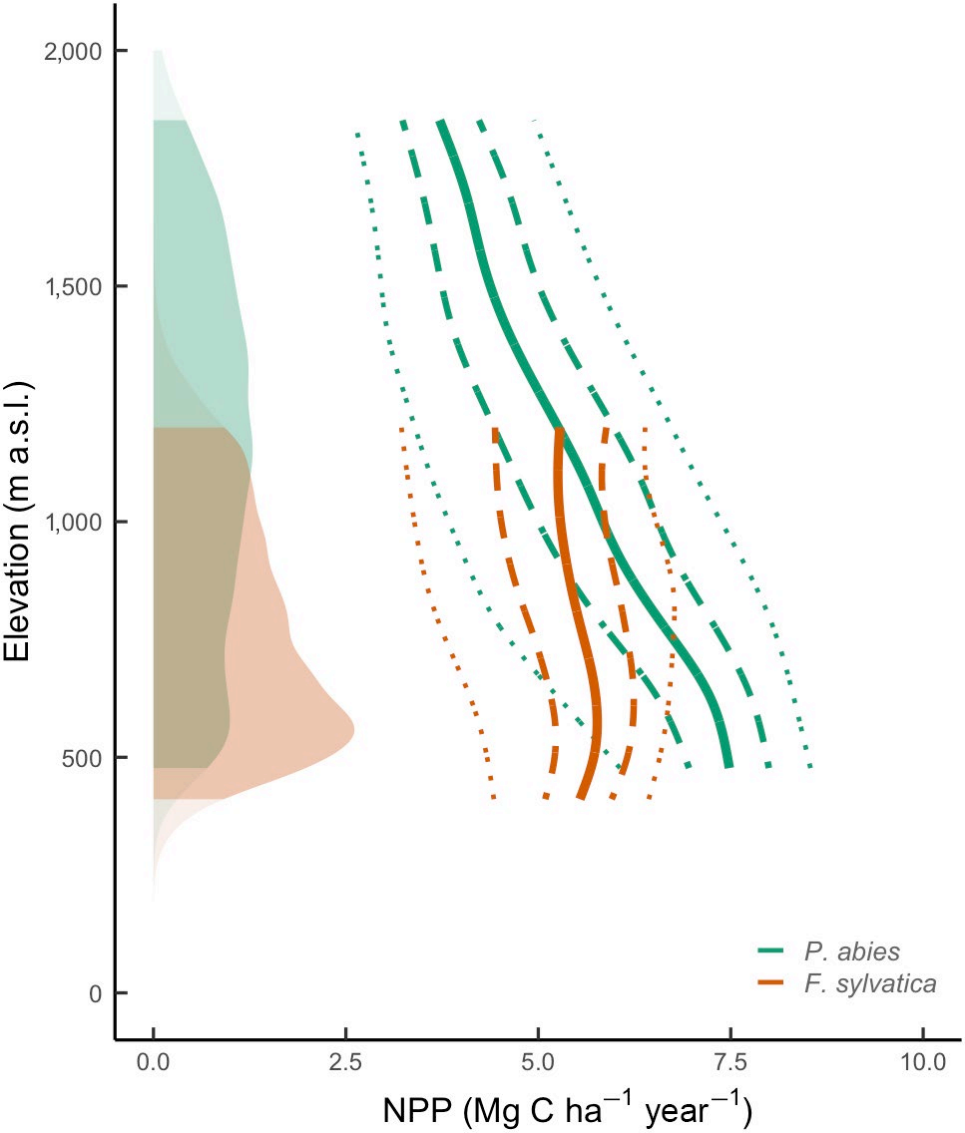
Trajectory of net primary productivity (NPP) along the elevational gradient simulated by the 3‐PG model for *Picea abies* (green) and *Fagus sylvatica* (orange) potential distribution ranges. The respective solid lines represent the average for the 1991–2018 period, the dashed and dotted lines represent 50% and 95% confidence interval, respectively. The shaded areas represent the density distribution of the potential species habitat along the elevational gradient

Annual mean net primary productivity (NPP) simulated on the 1 × 1 km grid for the described hypothetical stands at the age of 41–70 years within the species distribution range across Switzerland during the 1991–2018 period was 5.4 ± 1.5 Mg C ha^−1^ year^−1^ (mean ± standard deviation of the mean) for *P. abies* and 5.3 ± 1.0 Mg C ha^−1^ year^−1^ for *F. sylvatica* (Figure 3). There was a strong negative correlation between annual NPP and elevation (*p* < .001), with an average decrease of 2.86 ± 0.006 Mg C ha^−1^ year^−1^ km^−1^ for *P. abies* and 0.93 ± 0.010 Mg C ha^−1^ year^−1^ km^−1^ for *F. sylvatica*. On average, *P. abies* showed higher NPP (5.9 ± 4.1%) during the recent warmer period (1991–2018) compared to the reference period (1961–1990), while for *F. sylvatica*, the change was not significant. There was strong agreement in terms of the trend and the magnitude of NPP simulated by the 3‐PG model and NPP derived from MODIS (Figure S5) for *P. abies*, but less so for *F. sylvatica*.

**Figure 4:**
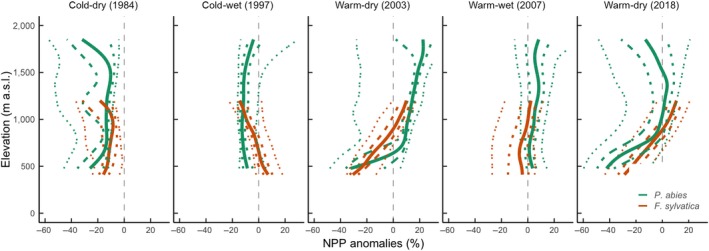
Trajectory of simulated net primary productivity (NPP) anomalies (percentage deviation) in selected extreme years (Figure S1) relative to the 1961–1990 reference period for *Picea abies* (green) and *Fagus sylvatica* (orange). The respective solid lines represent the average for the 1991–2018 period, the dashed and dotted lines represent 50% and 95% confidence interval, respectively. NPP anomalies were calculated for each grid cell of the potential species distribution ranges

**Figure 5:**
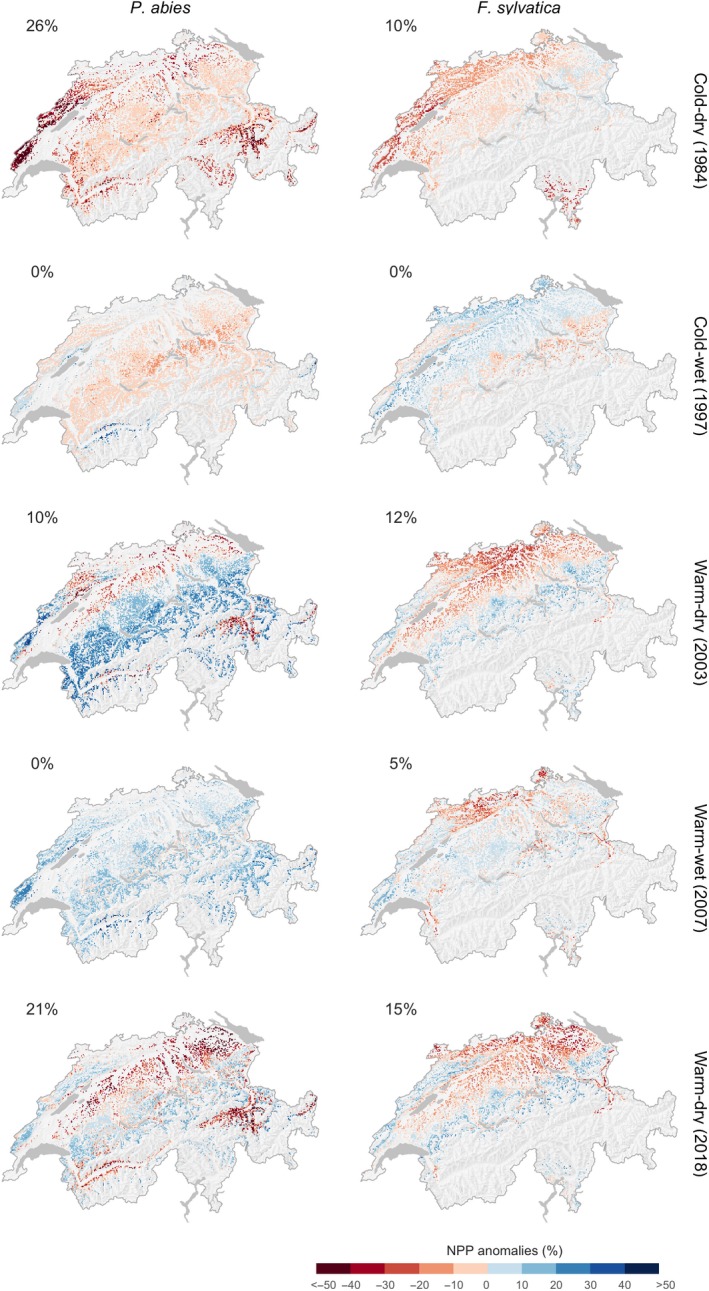
Spatial variation of simulated net primary productivity (NPP) anomalies (percent deviation) in selected extreme years relative to the 1961–1990 reference period for *Picea abies* (left) and *Fagus sylvatica* (right). Numbers indicate the percentage of grid cells across the potential species habitat that showed a strong negative response (> −25%)

The calibrated 3‐PG model indicates that annual NPP of *P. abies* and *F. sylvatica* was considerably reduced during extreme years (Figure 4). During the warm–dry year of 2018 (Figure S1), NPP was strongly reduced (anomaly below −25%) for one‐fifth of the potential habitat area in Switzerland (*P. abies*: 21%; *F. sylvatica*: 15%; Figure 5). Interestingly, the predictions for *P. abies* showed a different NPP anomaly in 2018 compared to the similarly warm–dry year of 2003, with NPP increasing at higher elevations in 2003 and NPP decreasing at lower elevations in both years. The reduction of NPP during the cold–dry extreme year of 1984 was comparable in extent and magnitude to the warm–dry year of 2018 (Figures 4 and 5).

## 4 DISCUSSION

Estimating the impact of climate extremes on forest ecosystem productivity is essential for understanding their role for regulating the regional carbon cycle and its drivers. Many previous studies have examined the effect of climate extremes on forests focus on extremely warm–dry years. We stress here the importance to also account for cold extremes, even though these might become less likely under climate change. By assimilating observations from 271 permanent long‐term forest monitoring plots with the 3‐PG forest ecosystem model, we were able to quantify the spatiotemporal changes in forest ecosystem productivity in response to climate extremes at the country scale, highlighting that not only extremely warm and dry years, but also extremely cold and/or wet years significantly impact forest NPP. Our results further indicated a high altitudinal and spatial variation in forest productivity response to climate extremes, which provides important information on forest vulnerability across the species' range.

### 4.1 Parameter estimation

So far, only few studies have assimilated extensive forest monitoring datasets into a DVM through techniques of parameter estimation (but see Cailleret et al., 2019; Fer et al., 2018; Minunno, Peltoniemi, et al., 2019; Thomas et al., 2017), even though recommended by several authors to improve large‐scale model projections (Dietze et al., 2014; Hartig et al., 2012). Our study demonstrates that it is possible to integrate monitoring data from multiple networks across a wide bioclimatic gradient into a process‐based forest ecosystem model 3‐PG. The resulting uncertainty in the parameter estimates was relatively low (Tables S3 and S4) and comparable to other studies that calibrated the 3‐PG model (Augustynczik et al., 2017; Thomas et al., 2017). Not surprisingly, the monitoring data were most informative for constraining parameters that are directly related to stand structure. However, the calibration also reduced parametric uncertainty in parameters not directly related to stand structure. For example, the maximum a posteriori estimates for the parameter *LAIgcx* (the LAI at which leaf area was not limiting transpiration) were reduced by 19% for *P. abies* and 5% for *F. sylvatica*, toward values consistent with empirical observations (Schulze, Kelliher, Korner, Lloyd, & Leuning, 1994). We conjecture that the lower reduction of parametric uncertainty in this parameter is both due to its lower influence on stand structure (to which the model was calibrated), but possibly also due to higher intraspecific variability, that is, the parameter values are not identical for all sites. To test this in future studies, spatially variable parameterization could be considered (cf. Vanderwel, Rozendaal, & Evans, 2017).

The relatively low predictive error of the calibrated model (e.g., pBias ≤ 9%) supports the use of Bayesian inference to estimate model parameters and their uncertainty. The observed reduction of the predictive error is comparable to that achieved in other studies in European forest landscapes (Minunno, Peltoniemi, et al., 2019; Van Oijen et al., 2013). The lower predictive error for *P. abies* compared to *F. sylvatica* (Figure 2) is likely due to the larger number of *P. abies* (*N* = 237) monitoring plots compared to *F. sylvatica* (*N* = 34) plots, especially due to the higher number of EFM plots (*P. abies*: 89; *F. sylvatica*: 5). In previous studies, data from permanent monitoring plots (like EFM) were shown to be more useful for model calibration than data from forest inventories (Minunno, Peltoniemi, et al., 2019; Van Oijen et al., 2013). Minunno, Peltoniemi, et al. (2019) argued that a main problem of the NFI data in their study was its shorter time span compared to their EFM data. The NFI and EFM data used by us have comparable time span, and might equally contribute to model calibration.

Even though our approach strongly improved model performance, we recognize and acknowledge some limitations. The reliability of initial conditions, climatic forcing data, and monitoring data are important for an ecologically meaningful data assimilation process (Van Oijen et al., 2013). Site nutrient status and available soil water are key variables in the 3‐PG model, but accurate in situ measurements are rare for a large number of plots. The soil suitability map that we used is rather generic and actual values for specific monitoring plots may differ substantially from the mapped data.

Moreover, as for the vast majority of other DGVMs, we assumed that species‐specific parameters are identical across their range, despite ample evidence for intraspecific variability of functional traits within species (Moran, Hartig, & Bell, 2016). Such a strong assumption simplifies the calibration process, but may also lead to inaccurate predictions about climate responses and forest resilience (see also Berzaghi et al., 2019). Thus, it will be beneficial to apply spatially variable parameterizations, despite the substantial computational cost. Thomas et al. (2017) successfully applied such an approach (Vanderwel et al., 2017) to constrain soil nutrient status in the 3‐PG model based on site index and mean annual temperature.

Finally, the question of model data assimilation is closely connected to structural model error. For example, in 3‐PG, the ratio between NPP and GPP is a constant, irrespective of environmental conditions (Amthor, 2000; DeLucia, Drake, Thomas, & Gonzalez‐Meler, 2007). Empirical studies, however, show that the GPP/NPP ratio can vary considerably (Collalti & Prentice, 2019; Zhang, Xu, Chen, & Adams, 2009). Another example is that inter‐annual variability in reproduction, not considered in 3‐PG, can substantially impact carbon use and forest growth, especially for masting species such as *F. sylvatica* (Hacket‐Pain et al., 2018). These and other limitations and uncertainties leave room for further improvement of 3‐PG and DVMs in general.

### 4.2 Simulations of NPP at the country scale

Simulated productivity for both species continuously decreased along the elevational gradient. This is consistent with results from other empirical studies in which forest productivity positively responds to temperature (Babst et al., 2013; Luyssaert et al., 2007). We also found strong agreement in the NPP–elevation relationship between 3‐PG and MODIS derived data for *P. abies*, but not for *F. sylvatica* (Figure S5). We think the latter is most likely due to the fact that the presence of *F. sylvatica* is firstly less accurately mapped than for *P. abies*, and secondly, that *F. sylvatica* often occurs in mixtures, making comparisons to MODIS data less reliable.

Simulated NPP across species distribution ranges was primarily controlled by temperature, soil water, and VPD (Figure S6). Environmental constraints due to low temperatures increased nonlinearly with increasing elevation, reflecting the temperature control of photosynthesis. Environmental constraints due to reduced soil water availability and increased VPD decreased with increasing elevation, but at a lower magnitude. Accordingly, optimal conditions for both *P. abies* and *F. sylvatica* were found at the lowest elevations. Surprisingly, the decrease in NPP along the elevational gradient for *F. sylvatica* is rather small compared to other studies (e.g., Zianis & Mencuccini, 2005). We hypothesize that this could be due to an incomplete coverage of the upper edge of the *F. sylvatica* distribution.

Our model simulated extreme years to cause substantial decreases in forest NPP along the Swiss elevational and bioclimatic gradient, which is in line with previous studies that assessed the impact of climate extremes on forest productivity during extremely warm–dry years (Kannenberg et al., 2019; Vitali, Büntgen, & Bauhus, 2017; Vitasse et al., 2019). Additionally, our study highlights the importance of accounting for both extreme cold and/or wet years. For both species, an extremely cold growing season can have a strong negative impact on NPP that is similar in magnitude to that from extreme warm–dry years (Figure 5). Similarly, Vitasse et al. (2019) found that late frosts can impact *F. sylvatica* growth in a magnitude comparable to extreme drought. However, it is also important to mention that 3‐PG and DGVMs in general have often overestimated the sensitivity of forest to drought, compared to observations (Klesse et al., 2018; Thomas et al., 2017).

Consistent with empirical studies, our analysis suggests that extreme years caused divergent forest growth responses along the Swiss elevational and spatial gradients (Hartl‐Meier, Dittmar, Zang, & Rothe, 2014; Jolly et al., 2005; Vitali et al., 2017). An increase in temperature can enhance growth at higher elevations but lead to drought‐induced growth decline at lower elevations, particularly for trees growing under high levels of competition (Babst et al., 2019; Jolly et al., 2005; Primicia et al., 2015; Schurman et al., 2019). As an example, the extremely warm–dry year of 2003 promoted better growing conditions at higher elevations for *P. abies*. At lower elevations, NPP decreased due to reduced soil water availability (caused by increasing evaporative demand) during the growing season (Figure S6). The year 2003 had a different pattern in precipitation than 2018, with smaller negative precipitation anomalies, especially at higher elevations (Figures S1 and S2). Thus, the abrupt decrease in NPP at lower elevations was compensated by an increase in NPP at higher elevations, which was not the case in 2018. For *F. sylvatica*, the substantial reduction in NPP during both extremely warm–dry years (2003 and 2018) was consistent with the patterns along the elevational and spatial gradients (Figures 4 and 5). The difference in precipitation anomalies between 2003 and 2018 occurred only above 1,200 m a.s.l., which is above the simulated range of *F. sylvatica*.

Because of increasing frequency and intensity of warm–dry events due to climate change, our results suggest that *P. abies* and *F. sylvatica* will show a substantial reduction in NPP at the lower elevational band, up to 800 m a.s.l. This effect is exacerbated by the fact that the drought response along climatic gradients will likely be altered in a nonlinear way (Kannenberg et al., 2019). However, the impact of drought on tree performance and forest productivity strongly depends on its seasonal timing (Crimmins, Crimmins, Gerst, Rosemartin, & Weltzin, 2017; Vicente‐Serrano et al., 2013), calling for more research on intra‐annual tree growth and climate sensitivity. Still, significant reduction in NPP on a large area (up to 21% for *P. abies* and 15% for *F. sylvatica*) in drought years provides incentive to reconsider the current forest management strategies and favor more drought tolerant genotypes of present tree species (Fréjaville, Fady, Kremer, Ducousso, & Garzón, 2019), or alternative species at the lower elevations. Furthermore, reducing low‐temperature constraints without necessarily reducing the probability of damaging late frosts (due to advanced phenology; Ma, Huang, Hänninen, & Berninger, 2019), our results suggest that *F. sylvatica* may experience a stronger reduction in NPP and potentially increased mortality in the future.

## 5 CONCLUSIONS

We assimilated an extensive collection of data from 271 permanent monitoring sites into the 3‐PG forest ecosystem model, and then simulated the climate sensitivity of two dominant European tree species across Switzerland. For the first time, it could be shown at a high spatial resolution that climate extremes impact forest productivity in more complex ways than simply shifting the response upwards in elevation. Our model suggests on the one hand that, during extremely warm–dry years, forests at lower elevations will suffer from soil water deficit and increased evaporative water demand, whereas forests at higher elevations, where trees are growing still below their temperature optimum, will benefit from warmer conditions. On the other hand, for trees growing on intermediate and less extreme sites, the model indicated highly differentiated year by year responses depending on the respective combination of weather forcing. The model–data fusion approach used in this study allowed us to model highly site‐specific NPP from long‐term monitoring data. Such robust estimates of NPP are key for increasing our understanding of forests carbon dynamics under climate extremes. During the extremely cold and wet years, both species experienced strong reductions in NPP, which are comparable in magnitude to extremely warm and dry years. Neither of these broad effects, however, are linear or homogenous in space. The nonlinear shifts in NPP during extreme years along the elevational gradients indicate the value and necessity of spatially resolved analyses of the impacts of climate extremes and changes.

## References

[gcb15011-bib-0001] Allen, C. D. , Macalady, A. K. , Chenchouni, H. , Bachelet, D. , McDowell, N. , Vennetier, M. , … Cobb, N. (2010). A global overview of drought and heat‐induced tree mortality reveals emerging climate change risks for forests. Forest Ecology and Management, 259, 660–684. 10.1016/j.foreco.2009.09.001

[gcb15011-bib-0002] Almeida, A. C. , Landsberg, J. J. , & Sands, P. J. (2004). Parameterisation of 3‐PG model for fast‐growing *Eucalyptus grandis* plantations. Forest Ecology and Management, 193, 179–195. 10.1016/j.foreco.2004.01.029

[gcb15011-bib-0003] Amthor, J. S. (2000). The McCree–de Wit‐Penning de Vries‐Thornley respiration paradigms: 30 years later. Annals of Botany, 86, 1–20. 10.1006/anbo.2000.1175

[gcb15011-bib-0004] Anderson, J. , Hoar, T. , Raeder, K. , Liu, H. , Collins, N. , Torn, R. , & Avellano, A. (2009). The data assimilation research testbed: A community facility. Bulletin of the American Meteorological Society, 90, 1283–1296. 10.1175/2009BAMS2618.1

[gcb15011-bib-0005] Augustynczik, A. L. D. , Hartig, F. , Minunno, F. , Kahle, H.‐P. , Diaconu, D. , Hanewinkel, M. , & Yousefpour, R. (2017). Productivity of *Fagus sylvatica* under climate change – A Bayesian analysis of risk and uncertainty using the model 3‐PG. Forest Ecology and Management, 401, 192–206. 10.1016/j.foreco.2017.06.061

[gcb15011-bib-0006] Babst, F. , Bouriaud, O. , Poulter, B. , Trouet, V. , Girardin, M. P. , & Frank, D. C. (2019). Twentieth century redistribution in climatic drivers of global tree growth. Science Advances, 5, eaat4313 10.1126/sciadv.aat4313 30746436 PMC6357745

[gcb15011-bib-0007] Babst, F. , Poulter, B. , Trouet, V. , Tan, K. , Neuwirth, B. , Wilson, R. , … Frank, D. (2013). Site‐ and species‐specific responses of forest growth to climate across the European continent. Global Ecology and Biogeography, 22, 706–717. 10.1111/geb.12023

[gcb15011-bib-0008] Beer, C. , Reichstein, M. , Tomelleri, E. , Ciais, P. , Jung, M. , Carvalhais, N. , … Papale, D. (2010). Terrestrial gross carbon dioxide uptake: Global distribution and covariation with climate. Science, 329, 834–838. 10.1126/science.1184984 20603496

[gcb15011-bib-0009] Berzaghi, F. , Wright, I. J. , Kramer, K. , Oddou‐Muratorio, S. , Bohn, F. J. , Reyer, C. P. O. , … Hartig, F. (2019). Towards a new generation of trait‐flexible vegetation models. Trends in Ecology & Evolution. 10.1016/j.tree.2019.11.006 31882280

[gcb15011-bib-0010] Bigler, C. , & Bugmann, H. (2018). Climate‐induced shifts in leaf unfolding and frost risk of European trees and shrubs. Scientific Reports, 8, 9865 10.1038/s41598-018-27893-1 29959342 PMC6026133

[gcb15011-bib-0011] Brändli, U.‐B. (2010). Schweizerisches Landesforstinventar: Ergebnisse der dritten Erhebung: 2004–2006. Birmensdorf, Switzerland: Swiss Federal Research Institute WSL.

[gcb15011-bib-0012] Brassel, P. , & Lischke, H. (2001). Swiss National Forest Inventory: Methods and models of the second assessment. Birmensdorf, Switzerland: Swiss Federal Research Institute WSL.

[gcb15011-bib-0013] Brockerhoff, E. G. , Barbaro, L. , Castagneyrol, B. , Forrester, D. I. , Gardiner, B. , González‐Olabarria, J. R. , … Jactel, H. (2017). Forest biodiversity, ecosystem functioning and the provision of ecosystem services. Biodiversity and Conservation, 26, 3005–3035. 10.1007/s10531-017-1453-2

[gcb15011-bib-0014] Cailleret, M. , Bircher, N. , Hartig, F. , Hülsmann, L. , & Bugmann, H. (2019). Bayesian calibration of a growth‐dependent tree mortality model to simulate the dynamics of European temperate forests. Ecological Applications. 10.1002/eap.2021 31605557

[gcb15011-bib-0015] Charney, N. D. , Babst, F. , Poulter, B. , Record, S. , Trouet, V. M. , Frank, D. , … Evans, M. E. K. (2016). Observed forest sensitivity to climate implies large changes in 21st century North American forest growth. Ecology Letters, 19, 1119–1128. 10.1111/ele.12650 27434040

[gcb15011-bib-0016] Chen, W. , Zhu, D. , Huang, C. , Ciais, P. , Yao, Y. , Friedlingstein, P. , … Zeng, N. (2019). Negative extreme events in gross primary productivity and their drivers in China during the past three decades. Agricultural and Forest Meteorology, 275, 47–58. 10.1016/j.agrformet.2019.05.002

[gcb15011-bib-0017] Ciais, P. , Sabine, C. , Bala, G. , Bopp, L. , Brovkin, V. , Canadell, J. , … Heimann, M. (2014). Carbon and other biogeochemical cycles In Climate change 2013: The physical science basis. Contribution of Working Group I to the Fifth Assessment Report of the Intergovernmental Panel on Climate Change (pp. 465–570). Cambridge, UK: Cambridge University Press.

[gcb15011-bib-0018] Clark, D. A. , Brown, S. , Kicklighter, D. W. , Chambers, J. Q. , Thomlinson, J. R. , & Ni, J. (2001). Measuring net primary production in forests: Concepts and field methods. Ecological Applications, 11, 356–370. 10.1890/1051-0761(2001)011[0356:MNPPIF]2.0.CO;2

[gcb15011-bib-0019] Collalti, A. , & Prentice, I. C. (2019). Is NPP proportional to GPP? Waring's hypothesis twenty years on. Tree Physiology, 39(8), 1473–1483. 10.1093/treephys/tpz034 30924876

[gcb15011-bib-0020] Cramer, W. , Bondeau, A. , Woodward, F. I. , Prentice, I. C. , Betts, R. A. , Brovkin, V. , … Young‐Molling, C. (2001). Global response of terrestrial ecosystem structure and function to CO_2_ and climate change: Results from six dynamic global vegetation models. Global Change Biology, 7, 357–373. 10.1046/j.1365-2486.2001.00383.x

[gcb15011-bib-0021] Crimmins, T. M. , Crimmins, M. A. , Gerst, K. L. , Rosemartin, A. H. , & Weltzin, J. F. (2017). USA National Phenology Network's volunteer‐contributed observations yield predictive models of phenological transitions. PLoS ONE, 12, e0182919 10.1371/journal.pone.0182919 28829783 PMC5568737

[gcb15011-bib-0022] Cuny, H. E. , Fonti, P. , Rathgeber, C. B. K. , von Arx, G. , Peters, R. L. , & Frank, D. C. (2019). Couplings in cell differentiation kinetics mitigate air temperature influence on conifer wood anatomy. Plant, Cell & Environment, 42, 1222–1232. 10.1111/pce.13464 30326549

[gcb15011-bib-0023] DeLucia, E. H. , Drake, J. E. , Thomas, R. B. , & Gonzalez‐Meler, M. (2007). Forest carbon use efficiency: Is respiration a constant fraction of gross primary production? Global Change Biology, 13, 1157–1167. 10.1111/j.1365-2486.2007.01365.x

[gcb15011-bib-0024] Dietze, M. C. , Lebauer, D. S. , & Kooper, R. (2013). On improving the communication between models and data. Plant, Cell & Environment, 36, 1575–1585. 10.1111/pce.12043 23181765

[gcb15011-bib-0025] Dietze, M. C. , Serbin, S. P. , Davidson, C. , Desai, A. R. , Feng, X. , Kelly, R. , … Wang, D. (2014). A quantitative assessment of a terrestrial biosphere model's data needs across North American biomes. Journal of Geophysical Research: Biogeosciences, 119, 286–300. 10.1002/2013JG002392

[gcb15011-bib-0026] D'Orangeville, L. , Houle, D. , Duchesne, L. , Phillips, R. P. , Bergeron, Y. , & Kneeshaw, D. (2018). Beneficial effects of climate warming on boreal tree growth may be transitory. Nature Communications, 9, 3213 10.1038/s41467-018-05705-4 PMC6086880 30097584

[gcb15011-bib-0027] Etzold, S. , Ruehr, N. K. , Zweifel, R. , Dobbertin, M. , Zingg, A. , Pluess, P. , … Buchmann, N. (2011). The carbon balance of two contrasting mountain forest ecosystems in Switzerland: Similar annual trends, but seasonal differences. Ecosystems, 14, 1289–1309. 10.1007/s10021-011-9481-3

[gcb15011-bib-0028] Etzold, S. , Waldner, P. , Thimonier, A. , Schmitt, M. , & Dobbertin, M. (2014). Tree growth in Swiss forests between 1995 and 2010 in relation to climate and stand conditions: Recent disturbances matter. Forest Ecology and Management, 311, 41–55. 10.1016/j.foreco.2013.05.040

[gcb15011-bib-0029] Fer, I. , Kelly, R. , Moorcroft, P. R. , Richardson, A. D. , Cowdery, E. M. , & Dietze, M. C. (2018). Linking big models to big data: Efficient ecosystem model calibration through Bayesian model emulation. Biogeosciences, 15, 5801–5830. 10.5194/bg-15-5801-2018

[gcb15011-bib-0030] Fischer C., & Traub B. (Eds.). (2019). Swiss National Forest Inventory – Methods and models of the fourth assessment, managing forest ecosystems. Dordrecht, the Netherlands: Springer International Publishing.

[gcb15011-bib-0031] Forrester, D. I. , Ammer, C. H. , Annighöfer, P. J. , Avdagic, A. , Barbeito, I. , Bielak, K. , … Bravo‐Oviedo, A. (2017). Predicting the spatial and temporal dynamics of species interactions in *Fagus sylvatica* and *Pinus sylvestris* forests across Europe. Forest Ecology and Management, 405, 112–133. 10.1016/j.foreco.2017.09.029

[gcb15011-bib-0032] Forrester, D. I. , Nitzsche, J. , & Schmid, H. (2019). The Experimental Forest Management project: An overview and methodology of the long‐term growth and yield plot network. Birmensdorf, Switzerland: Swiss Federal Institute of Forest, Snow and Landscape Research WSL.

[gcb15011-bib-0033] Forrester, D. I. , Tachauer, I. H. H. , Annighoefer, P. , Barbeito, I. , Pretzsch, H. , Ruiz‐Peinado, R. , … Sileshi, G. W. (2017). Generalized biomass and leaf area allometric equations for European tree species incorporating stand structure, tree age and climate. Forest Ecology and Management, 396, 160–175. 10.1016/j.foreco.2017.04.011

[gcb15011-bib-0034] Forrester, D. I. , & Tang, X. (2016). Analysing the spatial and temporal dynamics of species interactions in mixed‐species forests and the effects of stand density using the 3‐PG model. Ecological Modelling, 319, 233–254. 10.1016/j.ecolmodel.2015.07.010

[gcb15011-bib-0035] Frei, E. , Voegt, U. , Flueckiger, R. , Brunner, H. , Schai, F. , & Haeberli, R. (1980). Bodeneignungskarte der Schweiz. Neuchâtel, Switzerland: Bundesamt für Statistik, Sektion Geoinformation.

[gcb15011-bib-0036] Fréjaville, T. , Fady, B. , Kremer, A. , Ducousso, A. , & Garzón, M. B. (2019). Inferring phenotypic plasticity and population responses to climate across tree species ranges using forest inventory data. Global Ecology and Biogeography, 28, 1259–1271. 10.1111/geb.12930

[gcb15011-bib-0037] Gelman, A. , & Rubin, D. B. (1992). Inference from iterative simulation using multiple sequences. Statistical Science, 7, 457–472. 10.1214/ss/1177011136

[gcb15011-bib-0038] Gupta, R. , & Sharma, L. K. (2019). The process‐based forest growth model 3‐PG for use in forest management: A review. Ecological Modelling, 397, 55–73. 10.1016/j.ecolmodel.2019.01.007

[gcb15011-bib-0039] Hacket‐Pain, A. J. , Ascoli, D. , Vacchiano, G. , Biondi, F. , Cavin, L. , Conedera, M. , … Zang, C. S. (2018). Climatically controlled reproduction drives interannual growth variability in a temperate tree species. Ecology Letters, 21, 1833–1844. 10.1111/ele.13158 30230201 PMC6446945

[gcb15011-bib-0040] Halbritter, A. H. , Alexander, J. M. , Edwards, P. J. , & Billeter, R. (2013). How comparable are species distributions along elevational and latitudinal climate gradients? Global Ecology and Biogeography, 22, 1228–1237. 10.1111/geb.12066

[gcb15011-bib-0041] Hartig, F. , Dislich, C. , Wiegand, T. , & Huth, A. (2014). Technical note: Approximate Bayesian parameterization of a process‐based tropical forest model. Biogeosciences, 11, 1261–1272. 10.5194/bg-11-1261-2014

[gcb15011-bib-0042] Hartig, F. , Dyke, J. , Hickler, T. , Higgins, S. I. , O'Hara, R. B. , Scheiter, S. , & Huth, A. (2012). Connecting dynamic vegetation models to data – An inverse perspective. Journal of Biogeography, 39, 2240–2252. 10.1111/j.1365-2699.2012.02745.x

[gcb15011-bib-0043] Hartig, F. , Minunno, F. , & Paul, S. (2019). BayesianTools: General‐purpose MCMC and SMC samplers and tools for Bayesian statistics. Retrieved from https://cran.r-project.org/web/packages/BayesianTools/index.html

[gcb15011-bib-0044] Hartl‐Meier, C. , Dittmar, C. , Zang, C. , & Rothe, A. (2014). Mountain forest growth response to climate change in the Northern Limestone Alps. Trees, 28, 819–829. 10.1007/s00468-014-0994-1

[gcb15011-bib-0045] Houser, P. R. , De Lannoy, G. J. M. , & Walker, J. P. (2010). Land surface data assimilation In Lahoz W., Khattatov B., & Menard R. (Eds.), Data assimilation: Making sense of observations (pp. 549–597). Berlin, Heidelberg: Springer.

[gcb15011-bib-0046] Huang, Y. , Gerber, S. , Huang, T. , & Lichstein, J. W. (2016). Evaluating the drought response of CMIP5 models using global gross primary productivity, leaf area, precipitation, and soil moisture data. Global Biogeochemical Cycles, 30, 1827–1846. 10.1002/2016GB005480

[gcb15011-bib-0047] Huang, Y. , Stacy, M. , Jiang, J. , Sundi, N. , Ma, S. , Saruta, V. , … Luo, Y. (2019). Realized ecological forecast through an interactive Ecological Platform for Assimilating Data (EcoPAD, v1.0) into models. Geoscientific Model Development, 12, 1119–1137. 10.5194/gmd-12-1119-2019

[gcb15011-bib-0048] Humphrey, V. , Zscheischler, J. , Ciais, P. , Gudmundsson, L. , Sitch, S. , & Seneviratne, S. I. (2018). Sensitivity of atmospheric CO_2_ growth rate to observed changes in terrestrial water storage. Nature, 560, 628–631. 10.1038/s41586-018-0424-4 30158603

[gcb15011-bib-0049] Jolly, W. M. , Dobbertin, M. , Zimmermann, N. E. , & Reichstein, M. (2005). Divergent vegetation growth responses to the 2003 heat wave in the Swiss Alps. Geophysical Research Letters, 32 10.1029/2005GL023252

[gcb15011-bib-0050] Jung, M. , Reichstein, M. , Schwalm, C. R. , Huntingford, C. , Sitch, S. , Ahlström, A. , … Zeng, N. (2017). Compensatory water effects link yearly global land CO_2_ sink changes to temperature. Nature, 541, 516–520. 10.1038/nature20780 28092919

[gcb15011-bib-0051] Kannenberg, S. A. , Novick, K. A. , Alexander, M. R. , Maxwell, J. T. , Moore, D. J. P. , Phillips, R. P. , & Anderegg, W. R. L. (2019). Linking drought legacy effects across scales: From leaves to tree rings to ecosystems. Global Change Biology, 25, 2978–2992. 10.1111/gcb.14710 31132225

[gcb15011-bib-0052] Keenan, T. F. , Carbone, M. S. , Reichstein, M. , & Richardson, A. D. (2011). The model–data fusion pitfall: Assuming certainty in an uncertain world. Oecologia, 167, 587 10.1007/s00442-011-2106-x 21901361

[gcb15011-bib-0053] Keenan, T. F. , Davidson, E. , Moffat, A. M. , Munger, W. , & Richardson, A. D. (2012). Using model‐data fusion to interpret past trends, and quantify uncertainties in future projections, of terrestrial ecosystem carbon cycling. Global Change Biology, 18, 2555–2569. 10.1111/j.1365-2486.2012.02684.x

[gcb15011-bib-0054] Klesse, S. , Babst, F. , Lienert, S. , Spahni, R. , Joos, F. , Bouriaud, O. , … Frank, D. C. (2018). A combined tree ring and vegetation model assessment of European forest growth sensitivity to interannual climate variability. Global Biogeochemical Cycles. 10.1029/2017GB005856

[gcb15011-bib-0055] Körner, C. , & Paulsen, J. (2004). A world‐wide study of high altitude treeline temperatures. Journal of Biogeography, 31, 713–732. 10.1111/j.1365-2699.2003.01043.x

[gcb15011-bib-0056] Lahoz, W. , Khattatov, B. , & Menard, R. (2010). Data assimilation: Making sense of observations. Berlin, Heidelberg: Springer.

[gcb15011-bib-0057] Landsberg, J. , Mäkelä, A. , Sievänen, R. , & Kukkola, M. (2005). Analysis of biomass accumulation and stem size distributions over long periods in managed stands of *Pinus sylvestris* in Finland using the 3‐PG model. Tree Physiology, 25, 781–792. 10.1093/treephys/25.7.781 15870048

[gcb15011-bib-0058] Landsberg, J. , & Sands, P. (2011). Chapter 9—The 3‐PG process‐based model In Terrestrial ecology, physiological ecology of forest production (pp. 241–282). Elsevier 10.1016/B978-0-12-374460-9.00009-3

[gcb15011-bib-0059] Landsberg, J. J. , & Waring, R. H. (1997). A generalised model of forest productivity using simplified concepts of radiation‐use efficiency, carbon balance and partitioning. Forest Ecology and Management, 95, 209–228. 10.1016/S0378-1127(97)00026-1

[gcb15011-bib-0060] Lange, K. L. , Little, R. J. A. , & Taylor, J. M. G. (1989). Robust statistical modeling using the distribution. Journal of the American Statistical Association, 84, 881–896. 10.1080/01621459.1989.10478852

[gcb15011-bib-0061] LeBauer, D. S. , Wang, D. , Richter, K. T. , Davidson, C. C. , & Dietze, M. C. (2013). Facilitating feedbacks between field measurements and ecosystem models. Ecological Monographs, 83, 133–154. 10.1890/12-0137.1

[gcb15011-bib-0062] Luo, Y. , Ogle, K. , Tucker, C. , Fei, S. , Gao, C. , Ladeau, S. L. , … Schimel, D. S. (2011). Ecological forecasting and data assimilation in a data‐rich era. Ecological Applications, 21, 1429–1442. 10.1890/09-1275.1 21830693

[gcb15011-bib-0063] Luyssaert, S. , Inglima, I. , Jung, M. , Richardson, A. D. , Reichstein, M. , Papale, D. , … Janssens, I. A. (2007). CO_2_ balance of boreal, temperate, and tropical forests derived from a global database. Global Change Biology, 13, 2509–2537. 10.1111/j.1365-2486.2007.01439.x

[gcb15011-bib-0064] Ma, Q. , Huang, J.‐G. , Hänninen, H. , & Berninger, F. (2019). Divergent trends in the risk of spring frost damage to trees in Europe with recent warming. Global Change Biology, 25, 351–360. 10.1111/gcb.14479 30338890

[gcb15011-bib-0065] MacBean, N. , Peylin, P. , Chevallier, F. , Scholze, M. , & Schürmann, G. (2016). Consistent assimilation of multiple data streams in a carbon cycle data assimilation system. Geoscientific Model Development, 9, 3569–3588. 10.5194/gmd-9-3569-2016

[gcb15011-bib-0066] Minunno, F. , Hartig, F. , & Trotsiuk, V. (2019). threePGN – A Fortran implementation of the 3PGN model for R. Retrieved from https://github.com/checcomi/threePGN-package

[gcb15011-bib-0067] Minunno, F. , Peltoniemi, M. , Härkönen, S. , Kalliokoski, T. , Makinen, H. , & Mäkelä, A. (2019). Bayesian calibration of a carbon balance model PREBAS using data from permanent growth experiments and national forest inventory. Forest Ecology and Management, 440, 208–257. 10.1016/j.foreco.2019.02.041

[gcb15011-bib-0068] Moran, E. V. , Hartig, F. , & Bell, D. M. (2016). Intraspecific trait variation across scales: Implications for understanding global change responses. Global Change Biology, 22, 137–150. 10.1111/gcb.13000 26061811

[gcb15011-bib-0069] Nemani, R. R. , Keeling, C. D. , Hashimoto, H. , Jolly, W. M. , Piper, S. C. , Tucker, C. J. , … Running, S. W. (2003). Climate‐driven increases in global terrestrial net primary production from 1982 to 1999. Science, 300, 1560–1563. 10.1126/science.1082750 12791990

[gcb15011-bib-0070] Niu, S. , Luo, Y. , Dietze, M. C. , Keenan, T. F. , Shi, Z. , Li, J. , & Stuart Chapin III, F. (2014). The role of data assimilation in predictive ecology. Ecosphere, 5, art65 10.1890/ES13-00273.1

[gcb15011-bib-0071] Nolè, A. , Law, B. E. , Magnani, F. , Matteucci, G. , Ferrara, A. , Ripullone, F. , & Borghetti, M. (2009). Application of the 3‐PGS model to assess carbon accumulation in forest ecosystems at a regional level. Canadian Journal of Forest Research, 39, 1647–1661. 10.1139/X09-077

[gcb15011-bib-0072] Peng, C. , Guiot, J. , Wu, H. , Jiang, H. , & Luo, Y. (2011). Integrating models with data in ecology and palaeoecology: Advances towards a model–data fusion approach. Ecology Letters, 14, 522–536. 10.1111/j.1461-0248.2011.01603.x 21366814

[gcb15011-bib-0073] Peylin, P. , Bacour, C. , MacBean, N. , Leonard, S. , Rayner, P. , Kuppel, S. , … Prunet, P. (2016). A new stepwise carbon cycle data assimilation system using multiple data streams to constrain the simulated land surface carbon cycle. Geoscientific Model Development, 9, 3321–3346. 10.5194/gmd-9-3321-2016

[gcb15011-bib-0074] Piao, S. , Nan, H. , Huntingford, C. , Ciais, P. , Friedlingstein, P. , Sitch, S. , … Chen, A. (2014). Evidence for a weakening relationship between interannual temperature variability and northern vegetation activity. Nature Communications, 5, 5018 10.1038/ncomms6018 25318638

[gcb15011-bib-0075] Pretzsch, H. , Forrester, D. I. , & Rötzer, T. (2015). Representation of species mixing in forest growth models. A review and perspective. Ecological Modelling, 313, 276–292. 10.1016/j.ecolmodel.2015.06.044

[gcb15011-bib-0076] Primicia, I. , Camarero, J. J. , Janda, P. , Čada, V. , Morrissey, R. C. , Trotsiuk, V. , … Svoboda, M. (2015). Age, competition, disturbance and elevation effects on tree and stand growth response of primary *Picea abies* forest to climate. Forest Ecology and Management, 354, 77–86. 10.1016/j.foreco.2015.06.034

[gcb15011-bib-0077] R Core Team . (2018). R: A language and environment for statistical computing. Vienna, Austria: R Foundation for Statistical Computing.

[gcb15011-bib-0078] Reichstein, M. , Bahn, M. , Ciais, P. , Frank, D. , Mahecha, M. D. , Seneviratne, S. I. , … Wattenbach, M. (2013). Climate extremes and the carbon cycle. Nature, 500, 287–295. 10.1038/nature12350 23955228

[gcb15011-bib-0079] Rollinson, C. R. , Liu, Y. , Raiho, A. , Moore, D. J. P. , McLachlan, J. , Bishop, D. A. , … Dietze, M. C. (2017). Emergent climate and CO_2_ sensitivities of net primary productivity in ecosystem models do not agree with empirical data in temperate forests of eastern North America. Global Change Biology, 23, 2755–2767. 10.1111/gcb.13626 28084043

[gcb15011-bib-0080] Running, S. , Mu, Q. , & Zhao, M. (2011). MOD17A3 MODIS/Terra Net Primary Production Yearly L4 Global 1 km SIN Grid V055. NASA EOSDIS Land Processes DAAC.

[gcb15011-bib-0081] Sands, P. J. , & Landsberg, J. J. (2002). Parameterisation of 3‐PG for plantation grown *Eucalyptus globulus* . Forest Ecology and Management, 163, 273–292. 10.1016/S0378-1127(01)00586-2

[gcb15011-bib-0082] Schaub, M. , Dobbertin, M. , Kräuchi, N. , & Dobbertin, M. K. (2011). Preface—long‐term ecosystem research: Understanding the present to shape the future. Environmental Monitoring and Assessment, 174, 1–2. 10.1007/s10661-010-1756-1 21049286

[gcb15011-bib-0083] Scholze, M. , Buchwitz, M. , Dorigo, W. , Guanter, L. , & Quegan, S. (2017). Reviews and syntheses: Systematic Earth observations for use in terrestrial carbon cycle data assimilation systems. Biogeosciences, 14, 3401–3429. 10.5194/bg-14-3401-2017

[gcb15011-bib-0084] Schulze, E.‐D. , Kelliher, F. M. , Korner, C. , Lloyd, J. , & Leuning, R. (1994). Relationships among maximum stomatal conductance, ecosystem surface conductance, carbon assimilation rate, and plant nitrogen nutrition: A global ecology scaling exercise. Annual Review of Ecology and Systematics, 25, 629–660. 10.1146/annurev.es.25.110194.003213

[gcb15011-bib-0085] Schurman, J. S. , Babst, F. , Björklund, J. , Rydval, M. , Bače, R. , Čada, V. , … Svoboda, M. (2019). The climatic drivers of primary Picea forest growth along the Carpathian arc are changing under rising temperatures. Global Change Biology, 25(9), 3136–3150. 10.1111/gcb.14721 31166643

[gcb15011-bib-0086] Seddon, A. W. R. , Macias‐Fauria, M. , Long, P. R. , Benz, D. , & Willis, K. J. (2016). Sensitivity of global terrestrial ecosystems to climate variability. Nature, 531, 229–232. 10.1038/nature16986 26886790

[gcb15011-bib-0087] Senf, C. , Pflugmacher, D. , Zhiqiang, Y. , Sebald, J. , Knorn, J. , Neumann, M. , … Seidl, R. (2018). Canopy mortality has doubled in Europe's temperate forests over the last three decades. Nature Communications, 9, 4978 10.1038/s41467-018-07539-6 PMC6255806 30478255

[gcb15011-bib-0088] Shestakova, T. A. , Voltas, J. , Saurer, M. , Berninger, F. , Esper, J. , Andreu‐Hayles, L. , … Gutiérrez, E. (2019). Spatio‐temporal patterns of tree growth as related to carbon isotope fractionation in European forests under changing climate. Global Ecology and Biogeography, 28, 1295–1309. 10.1111/geb.12933

[gcb15011-bib-0089] Sommerfeld, A. , Senf, C. , Buma, B. , D'Amato, A. W. , Després, T. , Díaz‐Hormazábal, I. , … Seidl, R. (2018). Patterns and drivers of recent disturbances across the temperate forest biome. Nature Communications, 9, 4355 10.1038/s41467-018-06788-9 PMC6195561 30341309

[gcb15011-bib-0090] Swiss Federal Statistical Office . (2000). Swiss soil suitability map. BFS GEOSTAT. Retrieved from https://www.bfs.admin.ch/bfs/de/home/dienstleistungen/geostat/geodaten-bundesstatistik/boden-nutzung-bedeckung-eignung/abgeleitete-und-andere-daten/bodeneignungskarte-schweiz.html

[gcb15011-bib-0091] ter Braak, C. J. F. , & Vrugt, J. A. (2008). Differential evolution markov chain with snooker updater and fewer chains. Statistics and Computing, 18, 435–446. 10.1007/s11222-008-9104-9

[gcb15011-bib-0092] Thimonier, A. , Pannatier, E. G. , Schmitt, M. , Waldner, P. , Walthert, L. , Schleppi, P. , … Kräuchi, N. (2010). Does exceeding the critical loads for nitrogen alter nitrate leaching, the nutrient status of trees and their crown condition at Swiss Long‐term Forest Ecosystem Research (LWF) sites? European Journal of Forest Research, 129, 443–461. 10.1007/s10342-009-0328-9

[gcb15011-bib-0093] Thomas, R. Q. , Brooks, E. B. , Jersild, A. L. , Ward, E. J. , Wynne, R. H. , Albaugh, T. J. , … Teskey, R. O. (2017). Leveraging 35 years of *Pinus taeda* research in the southeastern US to constrain forest carbon cycle predictions: Regional data assimilation using ecosystem experiments. Biogeosciences, 14, 3525–3547. 10.5194/bg-14-3525-2017

[gcb15011-bib-0094] Thornton, P. E. , Running, S. W. , & White, M. A. (1997). Generating surfaces of daily meteorological variables over large regions of complex terrain. Journal of Hydrology, 190, 214–251. 10.1016/S0022-1694(96)03128-9

[gcb15011-bib-0095] UNECE ICP FOrests Programme Co‐ordinating Centre (Ed.). (2016). Manual on methods and criteria for harmonized sampling, assessment, monitoring and analysis of the effects of air pollution on forests. Eberswalde, Germany: Thunen Institute of Forest Ecosystems.

[gcb15011-bib-0096] van Oijen, M. (2017). Bayesian methods for quantifying and reducing uncertainty and error in forest models. Current Forestry Reports, 3, 269–280. 10.1007/s40725-017-0069-9

[gcb15011-bib-0097] van Oijen, M. , Reyer, C. , Bohn, F. J. , Cameron, D. R. , Deckmyn, G. , Flechsig, M. , … Rammer, W. (2013). Bayesian calibration, comparison and averaging of six forest models, using data from Scots pine stands across Europe. Forest Ecology and Management, 289, 255–268. 10.1016/j.foreco.2012.09.043

[gcb15011-bib-0098] Vanderwel, M. C. , Rozendaal, D. M. A. , & Evans, M. E. K. (2017). Predicting the abundance of forest types across the eastern United States through inverse modelling of tree demography. Ecological Applications, 27, 2128–2141. 10.1002/eap.1596 28675670

[gcb15011-bib-0099] Vicente‐Serrano, S. M. , Gouveia, C. , Camarero, J. J. , Beguería, S. , Trigo, R. , López‐Moreno, J. I. , … Sanchez‐Lorenzo, A. (2013). Response of vegetation to drought time‐scales across global land biomes. Proceedings of the National Academy of Sciences of the United States of America, 110, 52–57. 10.1073/pnas.1207068110 23248309 PMC3538253

[gcb15011-bib-0100] Vitali, V. , Büntgen, U. , & Bauhus, J. (2017). Silver fir and Douglas fir are more tolerant to extreme droughts than Norway spruce in south‐western Germany. Global Change Biology, 23, 5108–5119. 10.1111/gcb.13774 28556403

[gcb15011-bib-0101] Vitasse, Y. , Bottero, A. , Cailleret, M. , Bigler, C. , Fonti, P. , Gessler, A. , … Wohlgemuth, T. (2019). Contrasting resistance and resilience to extreme drought and late spring frost in five major European tree species. Global Change Biology, 25, 3781–3792. 10.1111/gcb.14803 31436853

[gcb15011-bib-0102] Waring, R. H. , Landsberg, J. J. , & Williams, M. (1998). Net primary production of forests: A constant fraction of gross primary production? Tree Physiology, 18, 129–134. 10.1093/treephys/18.2.129 12651397

[gcb15011-bib-0103] Wüest, R. O. , Bergamini, A. , Bollmann, K. , & Baltensweiler, A. (2020). LiDAR data as a proxy for light availability improve distribution modelling of woody species. Forest Ecology and Management, 456, 117644 10.1016/j.foreco.2019.117644

[gcb15011-bib-0104] Yoda, K. (1963). Self‐thinning in overcrowded pure stands under cultivated and natural conditions (Intraspecific competition among higher plants. XI). Journal of the Institute of Polytechnics, Osaka City University. Series D, 14, 107–129.

[gcb15011-bib-0105] Zhang, Y. , Xu, M. , Chen, H. , & Adams, J. (2009). Global pattern of NPP to GPP ratio derived from MODIS data: Effects of ecosystem type, geographical location and climate. Global Ecology and Biogeography, 18, 280–290. 10.1111/j.1466-8238.2008.00442.x

[gcb15011-bib-0106] Zhang, Z. , Babst, F. , Bellassen, V. , Frank, D. , Launois, T. , Tan, K. , … Poulter, B. (2018). Converging climate sensitivities of european forests between observed radial tree growth and vegetation models. Ecosystems, 21, 410–425. 10.1007/s10021-017-0157-5

[gcb15011-bib-0107] Zianis, D. , & Mencuccini, M. (2005). Aboveground net primary productivity of a beech (*Fagus moesiaca*) forest: A case study of Naousa forest, northern Greece. Tree Physiology, 25, 713–722. 10.1093/treephys/25.6.713 15805091

[gcb15011-bib-0108] Zielis, S. , Etzold, S. , Zweifel, R. , Eugster, W. , Haeni, M. , & Buchmann, N. (2014). NEP of a Swiss subalpine forest is significantly driven not only by current but also by previous year's weather. Biogeosciences, 11, 1627–1635. 10.5194/bg-11-1627-2014

