# Supplementary material for: Assessing the response of forest productivity to climate extremes in Switzerland using model–data fusion
Source: Glob Chang Biol. 2020 Feb 18;26(4):2463–76. doi: 10.1111/gcb.15011 (PMC7154780; doi:10.1111/gcb.15011)
Supplement: Supplementary file 1 [file GCB-26-2463-s001.docx]

**Supplementary materials for**

Assessing the response of forest productivity to climate extremes in Switzerland using model-data fusion

Volodymyr Trotsiuk^*^, Florian Hartig, Maxime Cailleret, Flurin Babst, David I. Forrester, Andri Baltensweiler, Nina Buchmann, Harald Bugmann, Arthur Gessler, Mana Gharun, Francesco Minunno, Andreas Rigling, Brigitte Rohner, Jonas Stillhard, Esther Thuerig, Peter Waldner, Marco Ferretti, Werner Eugster, Marcus Schaub

*Corresponding author. Email: [volodymyr.trotsiuk@usys.ethz.ch](mailto:volodymyr.trotsiuk@usys.ethz.ch)

**Supplementary Figures**


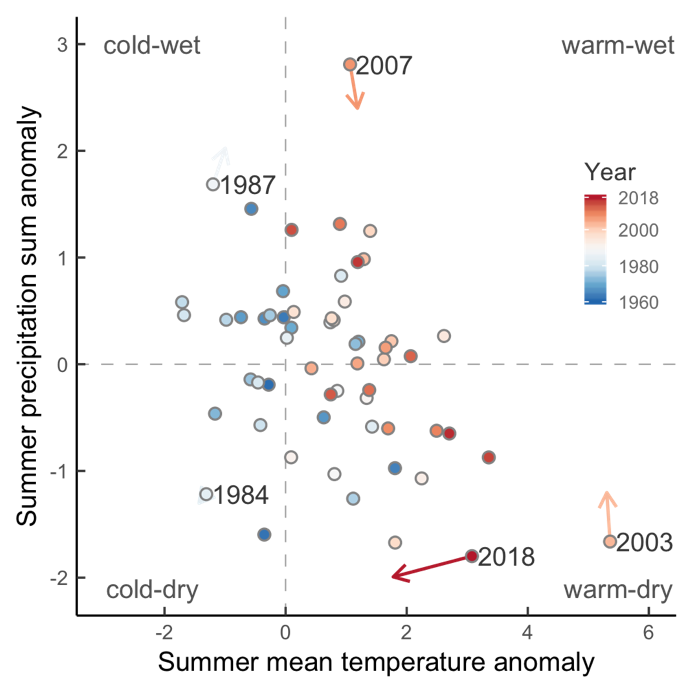


Figure S1. Growing season (May-August) mean temperature anomaly *vs.* growing season precipitation sum anomaly expressed by standard deviation compared to the reference period (1961-1990). Arrow lengths indicate the change in anomaly along the elevation gradient (per 1,000-m elevation change). E.g. year 2018 was less extreme in temperature in the upper elevations compared to the lower elevations.


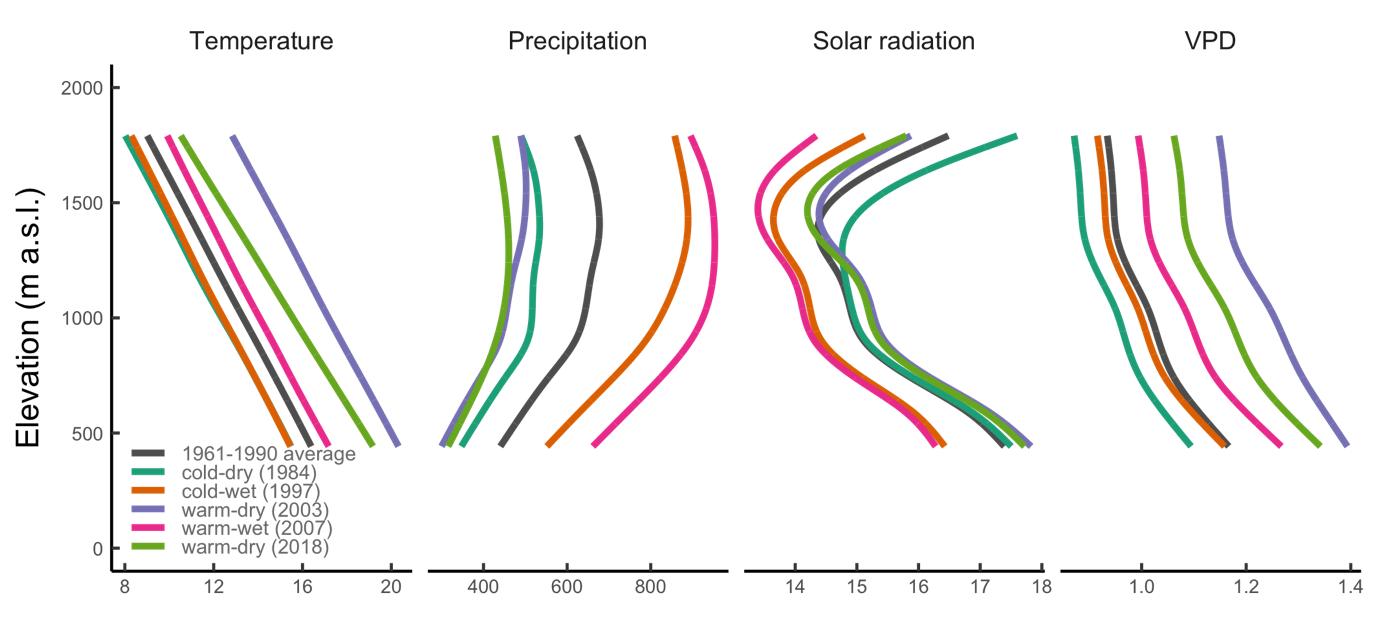


Fig S2. Values of main climatic forcing data along the elevation gradient. The black lines represent the average for the period (1961-1990). The extreme years are represented by colored lines. Values are calculated during the growing season (May-August): temperature – average (°C), precipitation – sum (mm), solar radiation – average (MJ m^-2^ d^-1^), vapor pressure deficit – average (kPa).

**
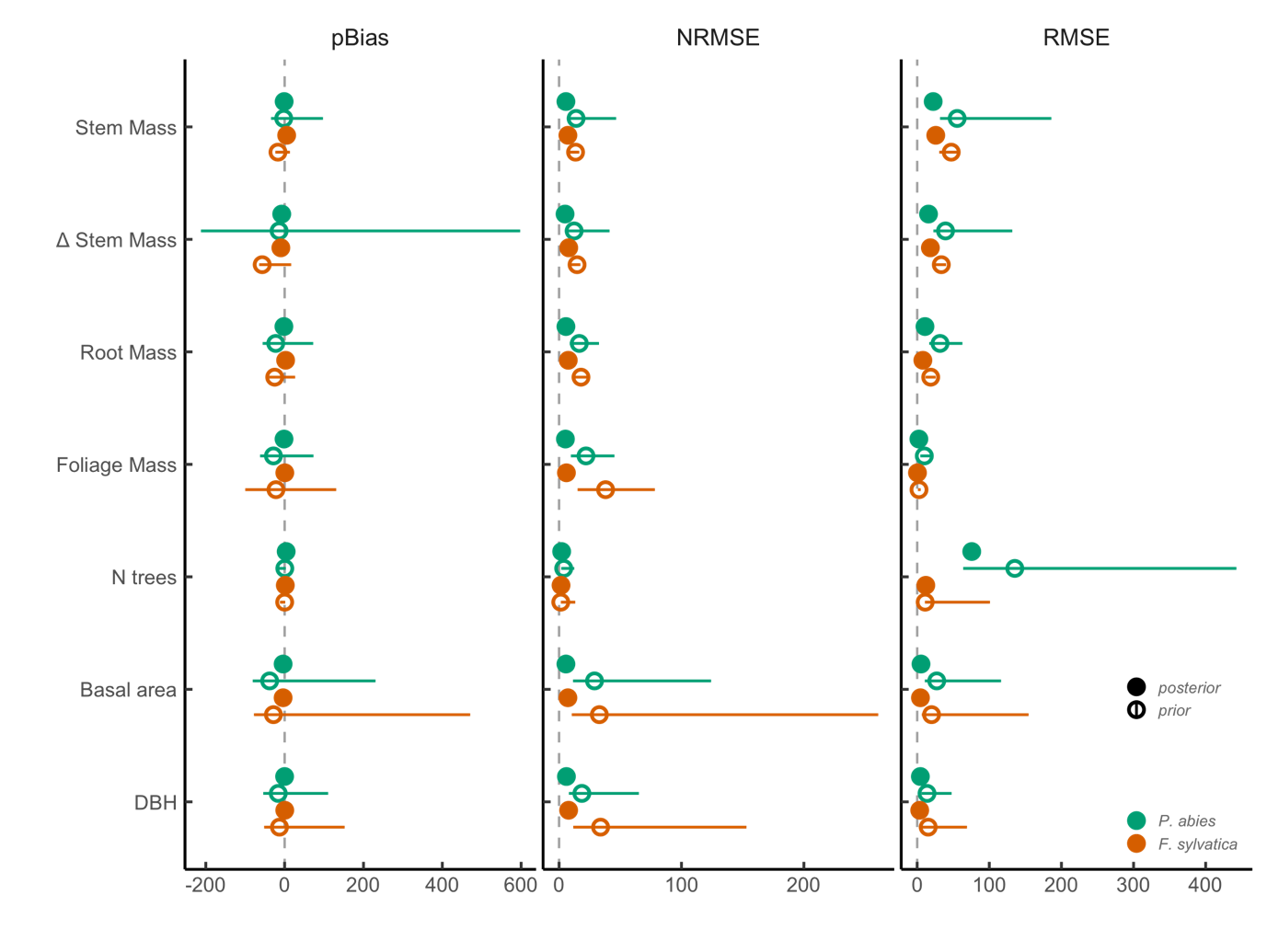
**

Fig S3. Statistics on predictive error (percent bias, normalized root mean squared error and root mean squared error) of the 3-PG model. The posterior predictive uncertainty was calculated by drawing 1,000 parameter combinations from the prior (empty dots) and posteriori (fill dots) distribution, and calculating model predictions for these combinations. The dots represent the median value of the posterior predictive distribution, while the horizontal lines represent the 95% confidence interval.

**
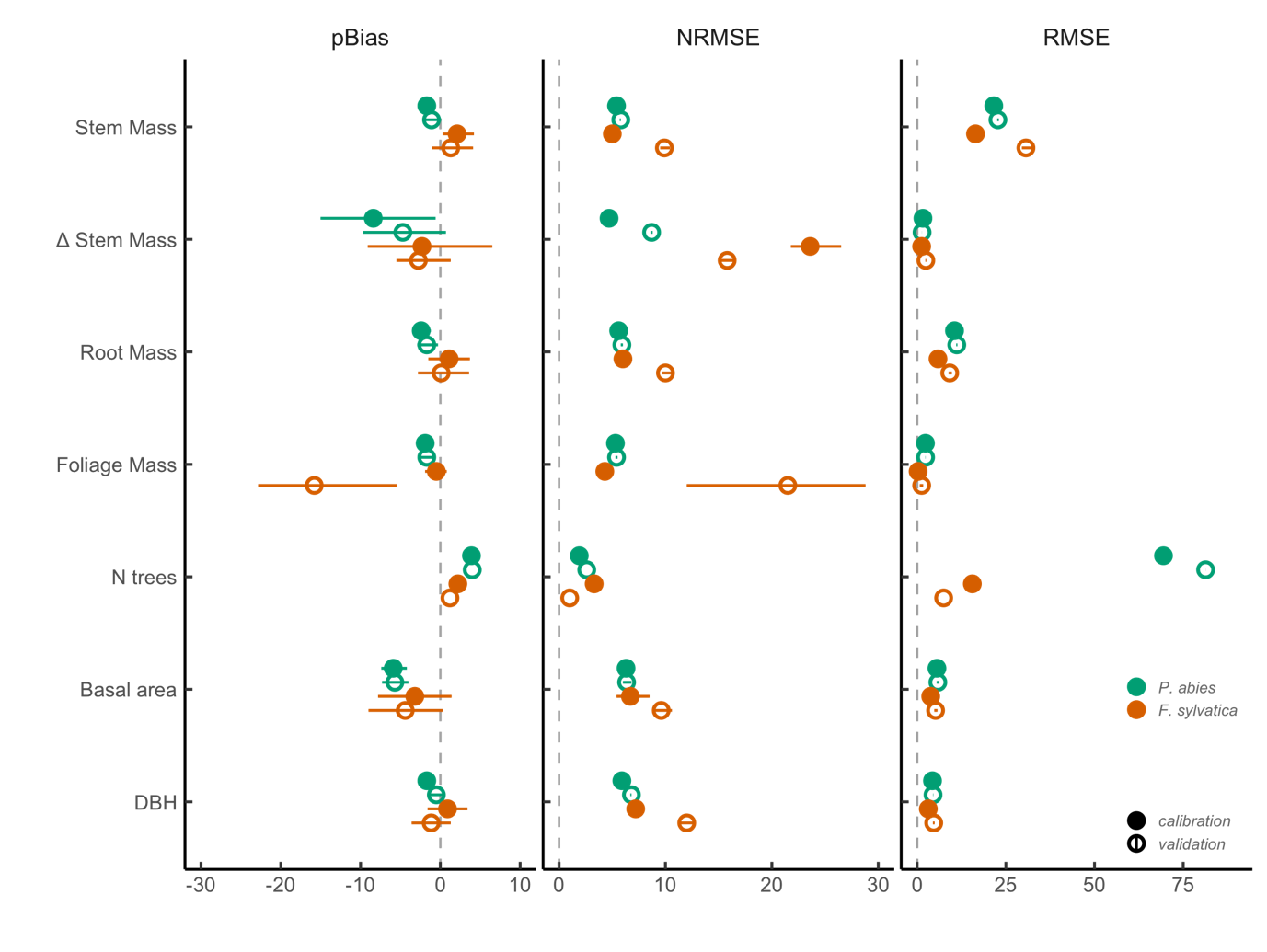
**

Fig S4. Statistics on predictive error (percent bias, normalized root mean squared error and root mean squared error) of the 3-PG model. The posterior predictive uncertainty was calculated by drawing 1,000 parameter combinations from the posteriori distribution, and calculating model predictions for the validation (empty dots) and calibration (fill dots) monitoring data subsets. The dots represent the median value of the posterior predictive distribution, while the horizontal lines represent the 95% confidence interval.


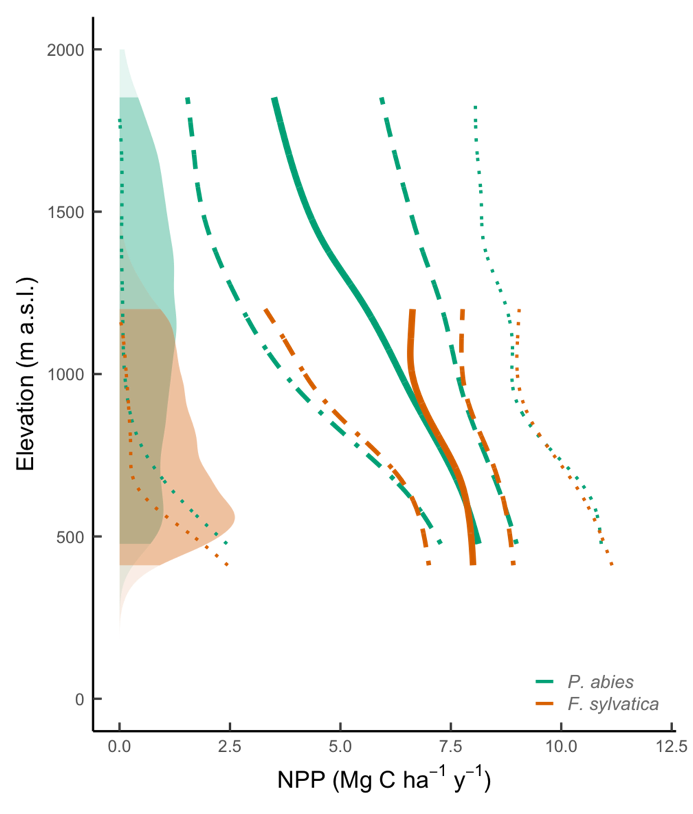


Figure S5. Trajectory of net primary productivity (NPP) along the elevational gradient derived from the MODIS (MOD17A3.055) for *P. abies* (green) and *F. sylvatica* (orange) potential distribution range. Potential habitats are based on the [MoGLI](https://www.wsl.ch/en/projects/mogli-modelling-woody-species-in-nfi.html) projections (Wüest et al., 2020). The respective solid lines represent the average for the 2000-2014 period, the dashed and dotted lines represent 50% and 95% confidence interval, respectively. The shaded areas represents the density distribution of the potential species habitat along the elevational gradient. Based on National Forest Inventory, only 57% (*P. abies*) and 32% (*F. sylvatica*) of the selected MODIS grid cells have a dominant species *P. abies* or *F. sylvatica,* respectively.

**
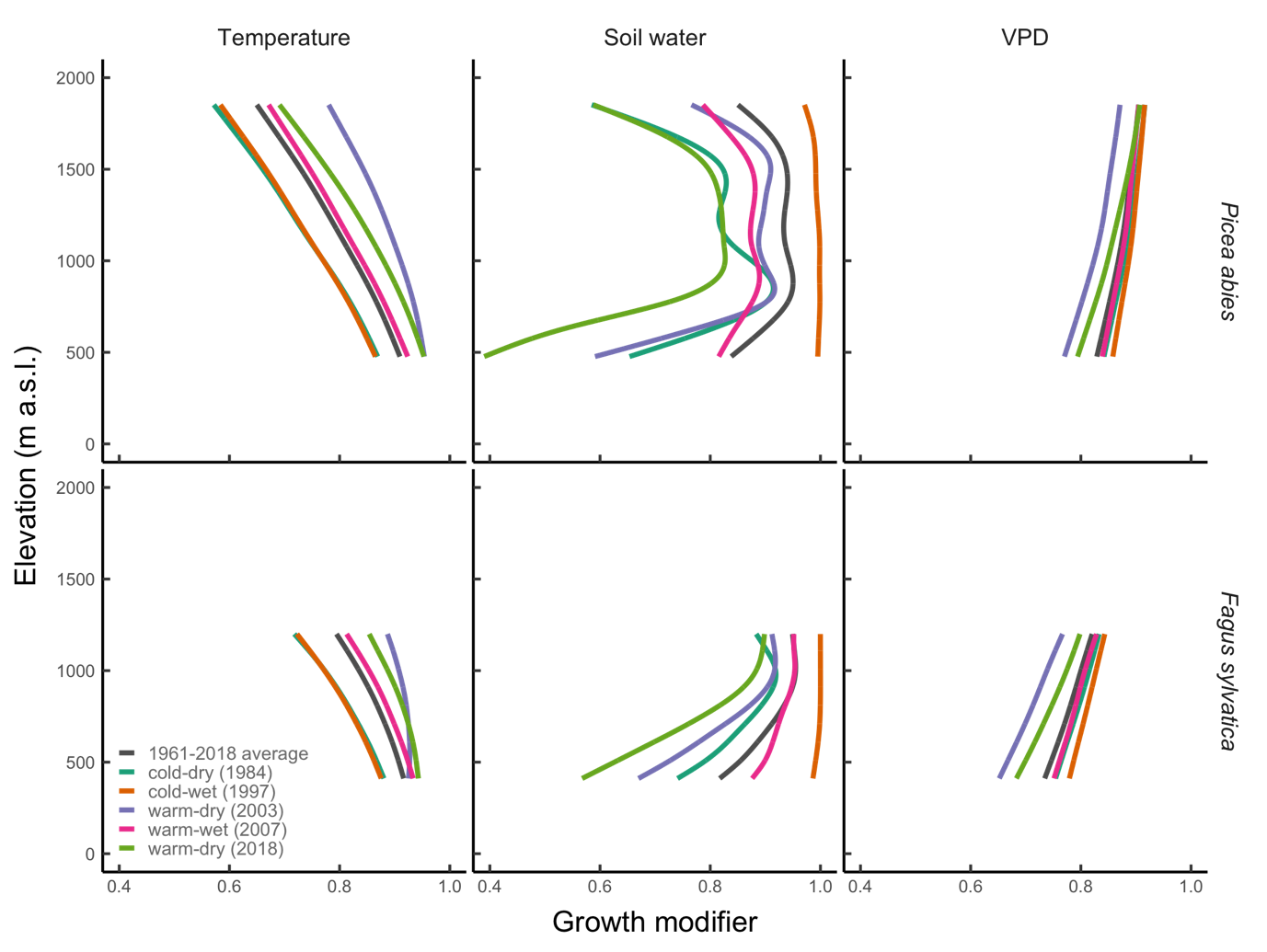
**

Fig S6. Changes in environmental modifiers along the elevation gradients simulated by the 3-PG model for *P. abies* (upper panel) and *F. sylvatica* (lower panel). The black lines represent the average for the period (1961-1990). The extreme years are represented by colored lines. Higher values (close to 1) of the growth modifier indicate less constraints on productivity.

**Supplementary Tables**

Table S1. Observational variables from four monitoring networks used for parameter estimation data assimilation.

| **ID** | **Abbreviation** | **Variable** | **Unit** | **Technique** |
| --- | --- | --- | --- | --- |
| 1 | Sb | Stem biomass | Mg ha^-1^ | Allometric equation |
| 2 | Rb | Root biomass | Mg ha^-1^ | Allometric equation |
| 3 | Fb | Foliage biomass | Mg ha^-1^ | Allometric equation |
| 4 | Ntrees | Number of trees per ha | number | Field measurement |
| 5 | BA | Basal area | m^2^ ha^-1^ | Field measurement |
| 6 | DBH | Average DBH | cm | Field measurement |
| 7 | LAI | Leaf area index | m^2^ m^-2^ | Allometric equation |
| 8 | GPP | Gross primary production | Mg C ha^-1^ m^-1^ | Eddy-covariance |

Table S2. Description of each parameter for which a posterior range was estimated.

| **Parameter** | **Description** | **Parameter** | **Description** |
| --- | --- | --- | --- |
| *Allometric relationships & partitioning* | | *Light interception* | |
| pFS2 | Foliage:stem partitioning ratio @ D=2 cm | K | Extinction coefficient for absorption of PAR by canopy |
| pFS20 | Foliage:stem partitioning ratio @ D=20 cm | fullCanAge | Age at canopy closure |
| aWS | Constant in the stem mass v. diam. Relationship | MaxIntcptn | Maximum proportion of rainfall evaporated from canopy |
| nWS | Power in the stem mass v. diam. Relationship | LAImaxIntcptn | LAI for maximum rainfall interception |
| pRx | Maximum fraction of NPP to roots | *Production and respiration* | |
| pRn | Minimum fraction of NPP to roots | alphaCx | Canopy quantum efficiency |
| *Litterfall & root turnover* | | Y | Ratio NPP/GPP |
| gammaF1 | Maximum litterfall rate | *Conductance* | |
| gammaF0 | Litterfall rate at t = 0 | MinCond | Minimum canopy conductance |
| tgammaF | Age at which litterfall rate has median value | MaxCond | Maximum canopy conductance |
| gammaR | Average monthly root turnover rate | LAIgcx | LAI for maximum canopy conductance |
| *Temperature modifier (fT)* | | CoeffCond | Defines stomatal response to VPD |
| Tmin | Minimum temperature for growth | Blcond | Canopy boundary layer conductance |
| Topt | Optimum temperature for growth | *Branch and bark fraction (fracBB)* | |
| Tmax | Maximum temperature for growth | fracBB0 | Branch and bark fraction at age 0 |
| *Soil water modifier (fSW)* | | fracBB1 | Branch and bark fraction for mature stands |
| Swconst | Moisture ratio deficit for fq = 0.5 | tBB | Age at which fracBB = (fracBB0+fracBB1)/2 |
| Swpower | Power of moisture ratio deficit | *Basic Density* | |
| *Atmospheric CO2 modifier (fCO2)* | | rhoMin | Minimum basic density – for young trees |
| fCalpha700 | Assimilation enhancement factor at 700 ppm | rhoMax | Maximum basic density – for older trees |
| fCg700 | Canopy conductance enhancement factor at 700 ppm | tRho | Age at which rho = (rhoMin+rhoMax)/2 |
| *Fertility effects* | | *Stem height* | |
| m0 | Value of ‘m’ when FR = 0 | aH | Constant in the stem height relationship |
| fN0 | Value of ‘fNutr’ when FR = 0 | nHB | Power of DBH in the stem height relationship |
| fNn | Power of (1-FR) in ‘fNutr’ | *Site information* | |
| *Age modifier (fAge)* | | Fr | Site fertility |
| MaxAge | Maximum stand age used in age modifier | *Standard deviation of observations* | |
| nAge | Power of relative age in function for fAge | sd_StemNo | Stem number standard deviation |
| rAge | Relative age to give fAge = 0.5 | sd_WS | Stem biomass standard deviation |
| *Stem mortality & self-thinning* | | sd_WF | Foliage biomass standard deviation |
| gammaN1 | Mortality rate for large t | sd_WR | Root biomass standard deviation |
| gammaN0 | Seedling mortality rate (t = 0) | sd_avDBH | Average DBH standard deviation |
| tgammaN | Age at which mortality rate has median value | sd_LAI | LAI standard deviation |
| ngammaN | Shape of mortality response | sd_GPP | GPP standard deviation |
| wSx1000 | Max. stem mass per tree @ 1000 trees/hectare | *Degrees of freedom parameter u* | |
| thinPower | Power in self-thinning rule | u_StemNo | Stem number degreed of freedom parameter u |
| mF | Fraction mean single-tree foliage biomass lost per dead tree | u_WS | Stem biomass degreed of freedom parameter u |
| mR | Fraction mean single-tree root biomass lost per dead tree | u_WF | Foliage biomass degreed of freedom parameter u |
| mS | Fraction mean single-tree stem biomass lost per dead tree | u_WR | Root biomass degreed of freedom parameter u |
| *Specific leaf area* | | u_avDBH | Average DBH degreed of freedom parameter u |
| SLA0 | Specific leaf area at age 0 | u_LAI | LAI degreed of freedom parameter u |
| SLA1 | Specific leaf area for mature leaves | u_GPP | GPP degreed of freedom parameter u |
| tSLA | Age at which specific leaf area = (SLA0+SLA1)/2 |  |  |

Table S3. Prior and posterior parameter estimates for P. abies, summarized by their quantiles. The reductions were calculated using the 95% of the posterior range.

| **Parameter** | **Prior range** | | **Posterior range** | | | **Reduction (%)** | **Convergence** | |
| --- | --- | --- | --- | --- | --- | --- | --- | --- |
|  | *min* | *max* | *2.50%* | *50.00%* | *97.50%* |  | *Point est.* | *Upper C.I.* |
| pFS2 | 0.3000 | 1.0000 | 0.3459 | 0.3795 | 0.4167 | 90 | 1.00 | 1.01 |
| pFS20 | 0.0300 | 0.3000 | 0.1319 | 0.1359 | 0.1401 | 97 | 1.01 | 1.01 |
| aWS | 0.0000 | 0.2500 | 0.0766 | 0.0836 | 0.0915 | 94 | 1.01 | 1.03 |
| nWS | 2.1000 | 2.8000 | 2.3450 | 2.3720 | 2.3983 | 92 | 1.01 | 1.03 |
| pRx | 0.3000 | 0.7000 | 0.3229 | 0.3477 | 0.3785 | 86 | 1.00 | 1.00 |
| pRn | 0.0100 | 0.4000 | 0.1349 | 0.1951 | 0.2220 | 78 | 1.00 | 1.00 |
| gammaF1 | 0.0001 | 0.0060 | 0.0001 | 0.0001 | 0.0001 | 100 | 1.00 | 1.00 |
| gammaF0 | 0.0001 | 0.0030 | 0.0001 | 0.0001 | 0.0001 | 99 | 1.00 | 1.01 |
| tgammaF | 12.0000 | 150.0000 | 20.2615 | 89.5449 | 146.6202 | 8 | 1.00 | 1.00 |
| gammaR | 0.0001 | 0.0040 | 0.0001 | 0.0001 | 0.0001 | 99 | 1.00 | 1.01 |
| Tmin | -5.0000 | 5.0000 | -4.7604 | -2.3015 | 0.5921 | 46 | 1.01 | 1.02 |
| Topt | 10.0000 | 30.0000 | 18.3438 | 22.0291 | 27.8260 | 53 | 1.00 | 1.01 |
| Tmax | 25.0000 | 40.0000 | 26.4063 | 33.6043 | 39.4511 | 13 | 1.01 | 1.01 |
| SWconst | 0.1000 | 1.0000 | 0.1210 | 0.1897 | 0.2299 | 88 | 1.00 | 1.01 |
| SWpower | 1.0000 | 20.0000 | 7.8599 | 12.4032 | 16.9304 | 52 | 1.00 | 1.01 |
| fCalpha700 | 1.0000 | 1.9000 | 1.0333 | 1.4608 | 1.8705 | 7 | 1.00 | 1.00 |
| fCg700 | 0.0001 | 1.0000 | 0.3521 | 0.7328 | 0.9758 | 38 | 1.02 | 1.05 |
| m0 | 0.0001 | 0.0300 | 0.0010 | 0.0145 | 0.0288 | 7 | 1.00 | 1.01 |
| fN0 | 0.0001 | 1.0000 | 0.1077 | 0.5656 | 0.9538 | 15 | 1.00 | 1.01 |
| fNn | 0.0001 | 2.0000 | 0.6950 | 1.5268 | 1.9733 | 36 | 1.00 | 1.01 |
| MaxAge | 200.0000 | 500.0000 | 239.1654 | 335.8899 | 464.3545 | 25 | 1.00 | 1.00 |
| nAge | 1.0000 | 4.3250 | 1.0009 | 1.0204 | 1.0821 | 98 | 1.00 | 1.00 |
| rAge | 0.0001 | 1.4000 | 0.0711 | 0.0991 | 0.1326 | 96 | 1.01 | 1.01 |
| gammaN1 | 0.0001 | 1.0000 | 0.0001 | 0.0001 | 0.0001 | 100 | 1.01 | 1.02 |
| gammaN0 | 0.0001 | 0.0300 | 0.0001 | 0.0001 | 0.0001 | 100 | 1.00 | 1.01 |
| tgammaN | 20.0000 | 80.0000 | 20.2700 | 25.9360 | 52.3079 | 47 | 1.01 | 1.01 |
| ngammaN | 0.0001 | 1.5000 | 0.1259 | 1.0416 | 1.4715 | 10 | 1.01 | 1.01 |
| wSx1000 | 300.0000 | 500.0000 | 457.6813 | 478.5689 | 498.6536 | 80 | 1.01 | 1.01 |
| thinPower | 1.0000 | 2.1000 | 1.0515 | 1.0978 | 1.1367 | 92 | 1.00 | 1.01 |
| mF | 0.0001 | 0.4000 | 0.0368 | 0.2473 | 0.3670 | 17 | 1.00 | 1.01 |
| mR | 0.0001 | 0.2500 | 0.0063 | 0.1051 | 0.2391 | 7 | 1.01 | 1.01 |
| mS | 0.0001 | 0.2500 | 0.0840 | 0.2018 | 0.2477 | 35 | 1.00 | 1.01 |
| SLA0 | 1.0000 | 15.0000 | 1.5711 | 4.2318 | 13.6533 | 14 | 1.00 | 1.01 |
| SLA1 | 1.0000 | 15.0000 | 4.0895 | 4.1238 | 4.1528 | 100 | 1.00 | 1.01 |
| tSLA | 3.0000 | 50.0000 | 3.2240 | 6.8364 | 22.7679 | 58 | 1.00 | 1.01 |
| k | 0.3500 | 0.6000 | 0.3504 | 0.3595 | 0.3803 | 88 | 1.00 | 1.01 |
| fullCanAge | 10.0000 | 50.0000 | 19.3680 | 21.5981 | 22.6350 | 92 | 1.00 | 1.00 |
| MaxIntcptn | 0.0001 | 0.4000 | 0.1410 | 0.2009 | 0.2551 | 71 | 1.00 | 1.01 |
| LAImaxIntcptn | 0.0001 | 10.0000 | 0.1326 | 2.5334 | 6.3681 | 38 | 1.00 | 1.00 |
| alphaCx | 0.0100 | 0.0900 | 0.0569 | 0.0696 | 0.0832 | 67 | 1.00 | 1.00 |
| Y | 0.4400 | 0.5100 | 0.4889 | 0.5038 | 0.5090 | 71 | 1.00 | 1.00 |
| MinCond | 0.0001 | 0.0200 | 0.0002 | 0.0031 | 0.0179 | 11 | 1.00 | 1.01 |
| MaxCond | 0.0001 | 0.0300 | 0.0149 | 0.0244 | 0.0297 | 50 | 1.00 | 1.01 |
| LAIgcx | 2.0000 | 5.0000 | 2.5047 | 4.2576 | 4.9474 | 19 | 1.01 | 1.01 |
| CoeffCond | 0.0001 | 0.0400 | 0.0069 | 0.0287 | 0.0391 | 19 | 1.00 | 1.01 |
| BLcond | 0.0001 | 0.1000 | 0.0008 | 0.0013 | 0.0018 | 99 | 1.00 | 1.00 |
| fracBB0 | 0.1000 | 1.0000 | 0.1373 | 0.5859 | 0.9644 | 8 | 1.00 | 1.01 |
| fracBB1 | 0.0001 | 0.2000 | 0.0078 | 0.1041 | 0.1939 | 7 | 1.00 | 1.01 |
| tBB | 10.0000 | 40.0000 | 10.6182 | 24.0197 | 39.1075 | 5 | 1.00 | 1.01 |
| rhoMin | 0.2000 | 0.7000 | 0.2053 | 0.3038 | 0.5742 | 26 | 1.00 | 1.01 |
| rhoMax | 0.2000 | 0.8000 | 0.2795 | 0.5320 | 0.7885 | 15 | 1.01 | 1.02 |
| tRho | 10.0000 | 150.0000 | 12.8099 | 51.3494 | 122.6771 | 22 | 1.01 | 1.02 |
| aH | 1.0000 | 6.0000 | 1.0971 | 2.8297 | 5.8060 | 6 | 1.00 | 1.01 |
| nHB | 0.0000 | 1.0000 | 0.0365 | 0.3600 | 0.9274 | 11 | 1.01 | 1.01 |
| fr | 0.0000 | 1.0000 | 0.3831 | 0.7679 | 0.9835 | 40 | 1.00 | 1.01 |
| sd_StemNo | 0.0010 | 15.0000 | 0.0104 | 0.0136 | 0.0178 | 100 | 1.00 | 1.01 |
| sd_WS | 0.0010 | 30.0000 | 11.0789 | 12.3608 | 13.7564 | 91 | 1.01 | 1.02 |
| sd_WF | 0.0010 | 5.0000 | 1.4838 | 1.6318 | 1.7883 | 94 | 1.01 | 1.02 |
| sd_WR | 0.0010 | 10.0000 | 5.0492 | 5.6928 | 6.2958 | 88 | 1.00 | 1.01 |
| sd_avDBH | 0.0010 | 10.0000 | 1.0759 | 1.2182 | 1.4455 | 96 | 1.01 | 1.01 |
| sd_LAI | 0.0010 | 5.0000 | 0.6078 | 0.6467 | 0.7308 | 98 | 1.00 | 1.01 |
| sd_GPP | 0.0010 | 5.0000 | 1.1028 | 1.4362 | 1.9294 | 83 | 1.00 | 1.01 |
| u_StemNo | 0.0001 | 1.0000 | 0.0001 | 0.0001 | 0.0001 | 100 | 1.01 | 1.02 |
| u_WS | 0.0001 | 1.0000 | 0.0230 | 0.0384 | 0.0501 | 97 | 1.01 | 1.02 |
| u_WF | 0.0001 | 1.0000 | 0.0372 | 0.0548 | 0.0867 | 95 | 1.00 | 1.01 |
| u_WR | 0.0001 | 1.0000 | 0.0187 | 0.0306 | 0.0432 | 98 | 1.01 | 1.01 |
| u_avDBH | 0.0001 | 1.0000 | 0.0003 | 0.0028 | 0.0071 | 99 | 1.00 | 1.01 |
| u_LAI | 0.0001 | 1.0000 | 0.0404 | 0.0609 | 0.0927 | 95 | 1.00 | 1.01 |
| u_GPP | 0.0001 | 1.0000 | 0.0093 | 0.0127 | 0.0184 | 98 | 1.00 | 1.01 |

Table S4. The prior and posterior parameter estimates for F. sylvatica, summarized by their quantiles. The reductions were calculated using the 95% of the posterior range.

| **Parameter** | **Prior range** | | **Posterior range** | | | **Reduction (%)** | **Convergence** | |
| --- | --- | --- | --- | --- | --- | --- | --- | --- |
|  | *min* | *max* | *2.50%* | *50.00%* | *97.50%* |  | *Point est.* | *Upper C.I.* |
| pFS2 | 0.3000 | 1.0000 | 0.3133 | 0.5381 | 0.9545 | 8 | 1.01 | 1.02 |
| pFS20 | 0.0300 | 0.3000 | 0.0325 | 0.0387 | 0.0466 | 95 | 1.00 | 1.01 |
| aWS | 0.0000 | 0.2500 | 0.0256 | 0.0468 | 0.0927 | 73 | 1.05 | 1.11 |
| nWS | 2.1000 | 2.8000 | 2.4416 | 2.6217 | 2.7792 | 52 | 1.04 | 1.09 |
| pRx | 0.3000 | 0.7000 | 0.3053 | 0.4381 | 0.6816 | 6 | 1.00 | 1.01 |
| pRn | 0.0001 | 0.4000 | 0.0509 | 0.1431 | 0.2102 | 60 | 1.01 | 1.02 |
| gammaF1 | 0.0001 | 0.0060 | 0.0001 | 0.0003 | 0.0008 | 90 | 1.02 | 1.03 |
| gammaF0 | 0.0001 | 0.0030 | 0.0001 | 0.0002 | 0.0005 | 86 | 1.02 | 1.04 |
| tgammaF | 12.0000 | 150.0000 | 16.4727 | 81.9099 | 146.1674 | 6 | 1.01 | 1.02 |
| gammaR | 0.0001 | 0.0040 | 0.0001 | 0.0003 | 0.0010 | 78 | 1.01 | 1.02 |
| Tmin | -5.0000 | 5.0000 | -4.7319 | -0.4167 | 4.4774 | 8 | 1.01 | 1.02 |
| Topt | 10.0000 | 30.0000 | 14.5507 | 20.3987 | 28.5519 | 30 | 1.02 | 1.05 |
| Tmax | 25.0000 | 40.0000 | 25.7761 | 33.5753 | 39.6549 | 7 | 1.01 | 1.01 |
| SWconst | 0.1000 | 1.2000 | 0.4589 | 1.0369 | 1.1926 | 33 | 1.03 | 1.06 |
| SWpower | 1.0000 | 13.0000 | 1.5087 | 7.3822 | 12.6696 | 7 | 1.00 | 1.01 |
| fCalpha700 | 1.0000 | 1.9000 | 1.0243 | 1.4482 | 1.8751 | 5 | 1.00 | 1.01 |
| fCg700 | 0.0001 | 1.0000 | 0.0291 | 0.4999 | 0.9719 | 6 | 1.01 | 1.02 |
| m0 | 0.0001 | 0.0300 | 0.0009 | 0.0154 | 0.0292 | 5 | 1.01 | 1.03 |
| fN0 | 0.0001 | 1.0000 | 0.0452 | 0.6027 | 0.9756 | 7 | 1.01 | 1.02 |
| fNn | 0.0001 | 2.0000 | 0.0936 | 1.0863 | 1.9451 | 7 | 1.02 | 1.04 |
| MaxAge | 200.0000 | 400.0000 | 207.8749 | 303.8503 | 394.7982 | 7 | 1.01 | 1.03 |
| nAge | 1.0000 | 4.3250 | 1.2596 | 2.9616 | 4.2334 | 11 | 1.01 | 1.02 |
| rAge | 0.0001 | 1.4000 | 0.6437 | 0.9972 | 1.3718 | 48 | 1.00 | 1.00 |
| gammaN1 | 0.0001 | 0.5000 | 0.0001 | 0.0001 | 0.0001 | 100 | 1.03 | 1.05 |
| gammaN0 | 0.0001 | 0.0300 | 0.0001 | 0.0001 | 0.0005 | 99 | 1.05 | 1.09 |
| tgammaN | 20.0000 | 80.0000 | 20.4245 | 32.6808 | 73.6895 | 11 | 1.03 | 1.05 |
| ngammaN | 0.0001 | 1.5000 | 0.1700 | 1.1566 | 1.4852 | 12 | 1.02 | 1.04 |
| wSx1000 | 150.0000 | 500.0000 | 150.8856 | 170.6656 | 230.1330 | 77 | 1.02 | 1.04 |
| thinPower | 1.0000 | 2.1000 | 1.4198 | 1.7388 | 1.8843 | 58 | 1.02 | 1.04 |
| mF | 0.0001 | 0.4000 | 0.0086 | 0.1748 | 0.3857 | 6 | 1.00 | 1.01 |
| mR | 0.0001 | 0.2500 | 0.0027 | 0.0588 | 0.2108 | 17 | 1.01 | 1.01 |
| mS | 0.0001 | 0.2500 | 0.0029 | 0.0738 | 0.2247 | 11 | 1.00 | 1.01 |
| SLA0 | 10.0000 | 30.0000 | 10.2444 | 15.0266 | 27.2618 | 15 | 1.02 | 1.04 |
| SLA1 | 10.0000 | 30.0000 | 19.8953 | 20.5913 | 21.2938 | 93 | 1.01 | 1.02 |
| tSLA | 3.0000 | 50.0000 | 5.7038 | 36.3405 | 49.4175 | 7 | 1.02 | 1.04 |
| k | 0.4000 | 0.6000 | 0.4005 | 0.4142 | 0.4639 | 68 | 1.02 | 1.04 |
| fullCanAge | 10.0000 | 50.0000 | 11.0415 | 31.2067 | 49.3648 | 4 | 1.00 | 1.00 |
| MaxIntcptn | 0.0001 | 0.4000 | 0.0130 | 0.2274 | 0.3916 | 5 | 1.01 | 1.02 |
| LAImaxIntcptn | 0.0001 | 10.0000 | 0.2915 | 5.1405 | 9.7417 | 5 | 1.01 | 1.02 |
| alphaCx | 0.0200 | 0.0600 | 0.0253 | 0.0375 | 0.0561 | 23 | 1.01 | 1.01 |
| Y | 0.4400 | 0.5100 | 0.4418 | 0.4736 | 0.5079 | 6 | 1.00 | 1.01 |
| MinCond | 0.0001 | 0.0200 | 0.0003 | 0.0053 | 0.0178 | 12 | 1.03 | 1.06 |
| MaxCond | 0.0001 | 0.0300 | 0.0032 | 0.0149 | 0.0286 | 15 | 1.03 | 1.06 |
| LAIgcx | 2.0000 | 4.0000 | 2.0505 | 2.9636 | 3.9442 | 5 | 1.01 | 1.01 |
| CoeffCond | 0.0001 | 0.0700 | 0.0082 | 0.0473 | 0.0687 | 13 | 1.01 | 1.02 |
| BLcond | 0.0001 | 0.1000 | 0.0021 | 0.0339 | 0.0953 | 7 | 1.01 | 1.02 |
| fracBB0 | 0.1000 | 1.0000 | 0.1247 | 0.5313 | 0.9709 | 6 | 1.01 | 1.01 |
| fracBB1 | 0.0001 | 0.2000 | 0.0063 | 0.1057 | 0.1950 | 6 | 1.02 | 1.03 |
| tBB | 10.0000 | 40.0000 | 10.9022 | 26.2373 | 39.1853 | 6 | 1.01 | 1.01 |
| rhoMin | 0.2000 | 0.7000 | 0.2072 | 0.3652 | 0.6535 | 11 | 1.01 | 1.02 |
| rhoMax | 0.2000 | 0.8000 | 0.3060 | 0.6049 | 0.7889 | 20 | 1.01 | 1.01 |
| tRho | 10.0000 | 150.0000 | 13.5932 | 77.9204 | 146.1178 | 5 | 1.01 | 1.03 |
| aH | 1.0000 | 6.0000 | 1.1414 | 3.4603 | 5.8505 | 6 | 1.00 | 1.01 |
| nHB | 0.0000 | 1.0000 | 0.0284 | 0.4858 | 0.9703 | 6 | 1.01 | 1.02 |
| fr | 0.0000 | 1.0000 | 0.1646 | 0.6605 | 0.9825 | 18 | 1.01 | 1.02 |
| sd_StemNo | 0.0010 | 15.0000 | 0.0018 | 0.0026 | 0.0038 | 100 | 1.01 | 1.01 |
| sd_WS | 0.0010 | 30.0000 | 18.7417 | 23.3649 | 28.5063 | 67 | 1.01 | 1.03 |
| sd_WF | 0.0010 | 5.0000 | 0.2808 | 0.3641 | 0.4553 | 97 | 1.01 | 1.02 |
| sd_WR | 0.0010 | 10.0000 | 5.5256 | 6.9510 | 8.7471 | 68 | 1.01 | 1.02 |
| sd_avDBH | 0.0010 | 10.0000 | 2.6370 | 3.3103 | 4.1001 | 85 | 1.01 | 1.02 |
| sd_LAI | 0.0010 | 5.0000 | 0.5682 | 0.7225 | 0.8988 | 93 | 1.01 | 1.01 |
| u_StemNo | 0.0001 | 1.0000 | 0.0001 | 0.0003 | 0.0009 | 100 | 1.01 | 1.02 |
| u_WS | 0.0001 | 1.0000 | 0.1041 | 0.5666 | 0.9743 | 13 | 1.01 | 1.02 |
| u_WF | 0.0001 | 1.0000 | 0.0551 | 0.4506 | 0.9642 | 9 | 1.00 | 1.01 |
| u_WR | 0.0001 | 1.0000 | 0.0706 | 0.4605 | 0.9644 | 11 | 1.01 | 1.02 |
| u_avDBH | 0.0001 | 1.0000 | 0.0789 | 0.5108 | 0.9702 | 11 | 1.01 | 1.01 |
| u_LAI | 0.0001 | 1.0000 | 0.0661 | 0.5141 | 0.9715 | 9 | 1.00 | 1.01 |

**Supplementary Code**

Code S1. Student’s t distribution likelihood function from Augustynczik et al. (2017)

$p\left( y|\theta\right)= \prod_{i=1}^{N} \frac{\Gamma(v+1)/2}{\Gamma(v/2)\sqrt{v\pi\sigma}}\left[ 1+ \frac{1}{v} \frac{1{(\hat{y}_{i}-y_{i})}^{2}}{v\sigma_{2}} \right]^{-(v+1)/2}$ (1)

where Γ: gamma function; ν: degrees of freedom of output variable; $y_{i}$: i^th^ observation of output variable; $\hat{y}_{i}$: i^th^ simulated value of output variable; and $\sigma_{i}^{2}$: variance of output variable. For the calibration, we parameterized $v$ (Eq. (2)) as

$v=1-N*ln(1-u)$ (2)

which maps the degrees of freedom from 1 to ∞, where N is a constant, related to the probability of having outliers in the dataset (Kruschke, 2014), defined in our case as 50. We then estimated the parameter $u$ with a uniform prior distribution from 0 to 1.

fLogL_Student <- function(sim, obs, s, u) {
 #' @description Student t log likelihood function. This function gives the likelihood value in logarithmic scale for a Student t distribution.
 #'
 #' @param sim vector of simulated data
 #' @param obs vector of observed data
 #' @param s numeric vector of standard deviations of the observed data
 #' @param u parameter to calculate degrees of freedom

 df <- 1 - 50 * log(1 - u)

 constant <- gamma( (df+1)/2) / ( sqrt(df*pi) * gamma(df/2) )

 Ri <- (sim - obs) / s

 logLi <- log(constant) - 0.5*log(s^2) - ((df+1) / 2 * log(1 + Ri^2/df) )

 sum( logLi )
}

**Literature Cited**

Augustynczik, A.L.D., Hartig, F., Minunno, F., Kahle, H.-P., Diaconu, D., Hanewinkel, M., Yousefpour, R., 2017. Productivity of Fagus sylvatica under climate change – A Bayesian analysis of risk and uncertainty using the model 3-PG. Forest Ecology and Management 401, 192–206. https://doi.org/10.1016/j.foreco.2017.06.061

Kruschke, J., 2014. Doing Bayesian data analysis: A tutorial with R, JAGS, and Stan. Academic Press.

Wüest, R.O., Bergamini, A., Bollmann, K., Baltensweiler, A., 2020. LiDAR data as a proxy for light availability improve distribution modelling of woody species. Forest Ecology and Management 456, 117644. https://doi.org/10.1016/j.foreco.2019.117644
